# An Early Triassic sauropterygian and associated fauna from South China provide insights into Triassic ecosystem health

**DOI:** 10.1038/s42003-020-0778-7

**Published:** 2020-02-11

**Authors:** Qiang Li, Jun Liu

**Affiliations:** 1 School of Resources and Environmental Engineering, Hefei University of Technology, Hefei, 230009 China; 2 Institute of Geosciences, University of Bonn, Bonn, 53115 Germany; 3 Nanjing Institute of Geology and Palaeontology, Nanjing, 210008 China

**Keywords:** Palaeontology, Phylogenetics, Palaeoecology

## Abstract

The timing and pattern of biotic recovery from the Permo-Triassic Mass Extinction remains elusive. Here we report new material of the Early Triassic sauropterygian *Lariosaurus sanxiaensis* and associated fauna from the Jialingjiang Formation in Hubei Province, South China. Phylogenetic analysis based on a novel data matrix of sauropterygians recognizes *L. sanxiaensis* as a basal nothosaur. Stratigraphic congruence analysis shows that the new phylogenetic consensus tree matches to the stratigraphic distribution of sauropterygians very well. The diversified reptilian fauna and inferred simple food web in the Nanzhang-Yuan’an fauna where *L. sanxiaensis* was discovered suggest that the Triassic biotic recovery adopted a top-down pattern, in contrast to the prevailing view. Comparison with the Middle Triassic Luoping biota from the same carbonate platform suggests that the Triassic biotic recovery is delayed and healthy ecosystems were not established until the Middle Triassic in South China.

## Abstract (web summary)

Qiang Li & Jun Liu present new fossils from the Early Triassic sauropterygian *Lariosaurus sanxiaensis* and associated reptile fauna recovered from South China. Their phylogenetic analysis places *L. sanxiaensis* as a basal nothosaur.

## Introduction

The timing and pattern of biotic recovery from the Permo-Triassic Mass Extinction (PTME), the largest catastrophe ever in earth history^1,2^, is controversial. The prevailing view maintains that the recovery was delayed at least until the Middle Triassic, and occurred in a step-wise pattern with low trophic-level groups recovering first^3^. In contrast to this prevailing model, extensive compilation of predators across the Permo-Triassic boundary shows that recovery had finished by the end of the Early Triassic^4–7^. Most recently, Song et al.^8^ proposed a top-down recovery from the PTME with a reversed trophic pyramid in the Early Triassic ecosystems and a normal pattern after the Middle Triassic based on an updated database of global fossil occurrences they compiled. They further suggested that taxonomic and ecological recoveries were decoupled, with taxonomic recovery finished by the beginning of Middle Triassic while ecological recovery was still underway until the end of the Triassic.

Accompanied with the recovery from the PTME is the origin of several entirely new groups of top marine predators in the early part of the Triassic, the sauropterygians (including placodonts and eosauropterygians, the latter of which is the inclusive group of plesiosaurs), ichthyosauromorphs and thalattosaurs^6,9–11^. Among Mesozoic marine reptiles, eosauropterygians are the most abundant group in terms of species diversity^12–14^. They originated in the Early Triassic, and played a major role in the Mesozoic oceanic ecosystem until their extinction at the end of Cretaceous^13^.

Triassic eosauropterygians can be broadly divided into three groups, the Pachypleurosauria (or pachypleurosaur-like forms sensu Cheng et al.^15^, which may be a paraphyletic group), the Nothosauroidea, and the Pistosauroidea^14^. Early Triassic eosauropterygians have been reported from both the Panthalassa and Tethyan provinces. They include the pachypleurosaur-like forms *Majiashanosaurus* and *Hanosaurus*, and the supposed pistosaur *Corosaurus*^6,16–18^. The earliest nothosaur, however, did not appear in the geological record until the early Middle Triassic when *Germanosaurus* and *Nothosaurus* were reported from Central Europe^14^. The existence of a ghost lineage of nothosaurs thus had to be hypothesized, as framed by the phylogenetic relationship of sauropterygians proposed by previous researchers^19–23^.

In this paper, we report a medium-sized nothosaur from the Lower Triassic Jialingjiang Formation, Yuan’an County, Hubei Province, China and discuss the implication of discovery of this earliest nothosaur on the phylogeny of eosauropterygians. Furthermore, we present the associated fauna where this early nothosaur was discovered and discuss the predator-prey relationship in this fauna. We aim to explore the food chain of the fauna and its implication to the biotic recovery from the PTME.

Institution Abbreviations: HFUT—Hefei University of Technology, Hefei, China; IVPP—Institute of Vertebrate Paleontology and Paleoanthropology, Beijing, China; WGSC—Wuhan Center of China Geological Survey, Wuhan, China; YIGM—Yichang Institute of Geology and Mineral Resources (now known as Wuhan Center of China Geological Survey), Yichang, China.

## Results

### Systematic paleontology

Reptilia Linnaeus, 1758

Sauropterygia Owen, 1840

Eosauropterygia Rieppel, 1994

Nothosauroidea Baur, 1889

*Lariosaurus sanxiaensis* Cheng in Chen et al.^24^.

### Holotype

YIGM V 0940, an articulated skeleton exposed in dorsal view, preserving part of the lower jaw, the cervical and the dorsal region.

### Referred specimen

HFUT YZS-16-01, an incomplete skeleton preserving posteriormost cervical, the dorsal, and part of the sacral skeleton.

### Horizon and locality

**Fig. 1:**
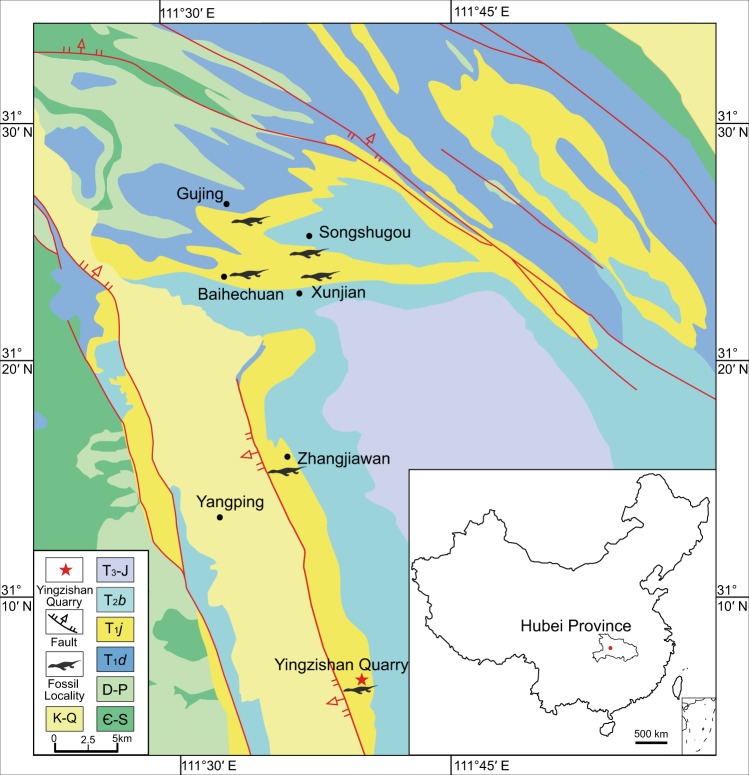
The brief geological map in the Nanzhang and Yuan’an counties showing the distribution of Triassic marine reptiles (updated after reference^59^ based on our field work). Inset is a map of China. Є-S Cambrian-Silurian; D-P Devonian-Permian; T_1_*d* Daye Formation, Lower Triassic; T_1_*j* Jialingjiang Formation, Lower Triassic; T_2_*b* Badong Formation, Middle Triassic; T_3_-J Upper Triassic-Jurassic; K-Q Cretaceous-Quaternary.

The Third Member of Jialingjiang Formation, Nanzhang and Yuan’an counties, Hubei Province. Lithological correlation and biostratigraphic investigation suggested a late Early Triassic age for the horizon^25^. The new material described here was collected from a limestone quarry in the southwestern side of Yingzishan hill, Yuan’an County, Hubei Province in 2008 (see Fig. 1). There are a series of limestone quarries along the western side of Yingzishan hill, but they have all been closed now for the restoration of forest in the region. Yingzishan was previously known as Wangchenkang when Young^26^ described *Keichousaurus yuananensis*. *K. yuananensis* was described as a pachypleurosaur, but the type specimen has been lost and its species validity has been questioned^14^.

### Revised diagnosis

A medium-sized nothosaur with much shortened cervical region; distinctly pachyostotic dorsal neural arches and ribs with single head; oval-like coracoid with a notch in the posterolateral margin; a distinctly small humerus; straight ulna with slight constriction in the middle; transverse processes approaching to the ventral margin of the corresponding centra.

### Systematic note

Cheng^27^ named *Lariosaurus sanxiaensis* and briefly described its holotype in his PhD thesis. The thesis was clearly indicated by the author (dated on 28 May 2015) to be treated as a publication in the CNKI database. So the thesis meets the criteria of electronic publication (ICZN Code Articles 8.1, 8.5.1 and 8.5.2). However, the species name does not meet the criteria of electronic publication since the name was not registered into the ZooBank, so the species name cannot be taken as officially published (ICZN Code Article 8.5.3). The species was named again by Cheng in Chen et al.^24^, and the name can be taken as officially published in this monograph.

### Overall description

The specimen described here preserves the posteriormost cervical, the dorsal, and part of the sacral skeleton. Most part of the skeleton is articulated. The specimen is preserved in gray micritic limestone, with a thin argillaceous matrix covering the bone. Measurements of the specimen are given in Table 1.

**Table 1:** Measurements of the referred specimen of *Lariosaurus sanxiaensis* (HFUT YZS-16-01).

Measurement	Value (mm)
Length of the right humerus	58.02
Proximal width of the right humerus	10.02
Distal width of the right humerus	19.1
Length of the ulna	37.85
Proximal width of the ulna	13.05
Distal width of the ulna	10.17
Standard length	70

### Vertebrae

HFUT YZS-16-01 preserves 30 vertebrae in total. All vertebrae were exposed in dorsal view prior to collection. The cervical region of HFUT YZS-16-01 is largely incomplete. We infer that the two most anteriorly preserved vertebrae are likely cervical vertebrae because of their position relative to the scapula and the intermediate length of the first dorsal rib compared with the relatively stable length of those dorsal ribs immediately posterior to it. The holotype of *Lariosaurus sanxiaensis* preserves a relatively complete cervical region and 12 cervical vertebrae have been estimated for it^27^. We infer that the two most posteriorly preserved vertebrae in HFUT YZS-16-01 are likely sacral vertebrae because of the significantly decreased length and curvature of the presumably two posteriormost dorsal ribs compared with those of other dorsal ribs. The pattern of such a dorsal rib length and shape change is common among Triassic eosauropterygians^14^. Thus we estimate that there are probably 26 dorsal vertebrae in total in *L. sanxiaensis*. However, we acknowledge the uncertainty of the estimation of both the last cervical vertebra and the first sacral vertebra, and the actual number of dorsal vertebrae in HFUT YZS-16-01 could be more considering the length variation pattern of the first and the last dorsal ribs among Triassic eosauropterygians.

The neural spines have mostly been weathered away in HFUT YZS-16-01, but it has been reported to be very low in the holotype. The centra are well articulated with each other. From the ventral view, all of the preserved centra have parallel edges. There is no subcentral foramen visible on the ventral surface of centra. Intercentra are also absent along the whole skeleton. Pachyostosis is notable in zygapophyses. If correctly identified, the posteriormost cervical centrum does not have a keeled ventral surface. However, a keeled ventral surface has been reported to be present in the anterior cervical centra of the holotype^27^.

The transverse processes of dorsal vertebrae are short. Vertebrae 12–14 were also prepared from the lateral side, showing elliptic cross section of the transverse processes without the increase of diameter toward the end. The location of transverse processes approaches to the ventral margin of the corresponding centra, which is an autapomorphy among Triassic sauropterygians.

### Ribs and gastralia

**Fig. 2:**
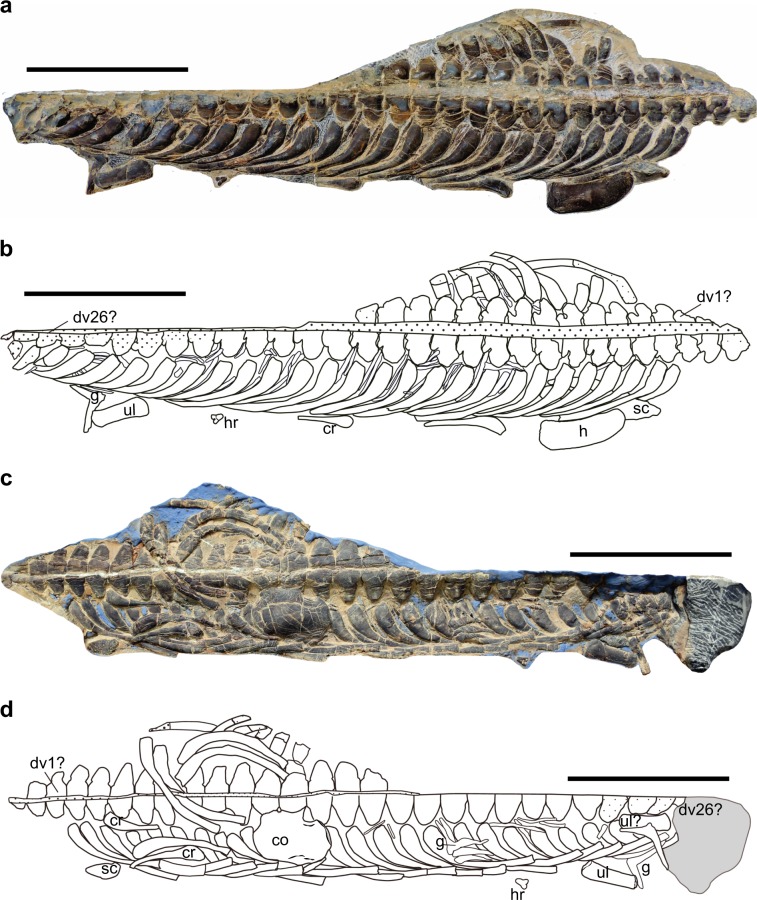
HFUT YZS-16-01. **a** Photo showing the dorsal view of the skeleton. **b** Interpretative drawing. **c** Photo showing the ventral view of the skeleton. **d** Interpretative drawing. co coracoid, cr cervical rib, dv dorsal vertebra, g gastralia, h humerus, ha hemal arch, sc scapula, ul ulna. Scale bars equal 10 cm.

All of the preserved ribs are distinctly pachyostotic. Judging from the shape and the length, there are at least three dislocated cervical ribs preserved (see Fig. 2). One of them is visible from the dorsal view (Fig. 2a).

All of the preserved cervical ribs are single-headed and do not have an anterior process. The rib head morphology seems different from the holotype. In the holotype, the preserved anterior cervical ribs (presumably 5th–11th) were all described as with an apparent anterior process^27^. This suggests that the shorter cervical rib likely belongs to one of the four anteriormost cervical ribs. From the ventral view, there are two presumably cervical ribs visible (see Fig. 2c, d). They are all single headed and no anterior process can be discerned. According to Cheng^27^, the cervical ribs of the holotype are double-headed, so the cervical ribs preserved here with a single head could be the last few cervical ribs that share similar morphology with dorsal ribs. The dorsal ribs are single-headed. No posterior groove of the dorsal ribs around the shoulder region can be observed.

**Fig. 3:**
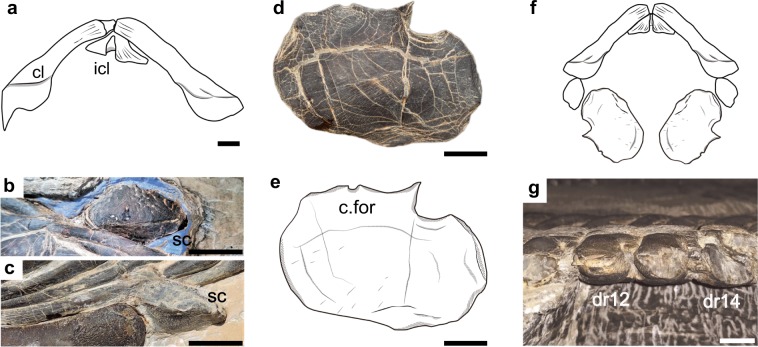
Pectoral girdle and vertebrae of *Lariosaurus sanxiaensis*. **a** Drawing of clavicle and interclavicle of the holotype (YIGM V 0940, redrawn after Cheng^27^). **b**, **c** Photograph of the scapula of HFUT YZS-16-01, in ventral and dorsal view respectively. **d**, **e** Photograph and drawing of the dislocated coracoid of HFUT YZS-16-01. **f** Reconstruction of the complete pectoral girdle of *Lariosaurus sanxiaensis* in ventral view. **g** The lateral view of the supposed 14th to 16th dorsal vertebra of HFUT YZS-16-01. c. for coracoid foramen, cl clavicle, icl interclavicle, sc scapula. Scale bars equal 1 cm.

In sauropterygians, the gastralia are well developed and closely approaching one another, spanning the entire dorsal region^14^. In HFUT YZS-16-01, several dislocated median gastral elements are preserved and closely interlocked with each other, showing an angular shape (Fig. 3a). All of the preserved median gastral elements have a single lateral process.

### Pectoral girdle

In the holotype, the interclavicle and clavicles are completely preserved (Fig. 3a) and part of the scapulae and coracoids are also preserved^27^. In HFUT YZS-16-01, the right scapula and coracoid of the pectoral girdle are completely preserved (Fig. 3b–e). All other pectoral girdle elements are lost. The clavicles meet each other in front of the interclavicle and become narrower medially in the holotype. The anterolateral corner of clavicles is not expanded. The interclavicle has a roughly triangular shape. The right scapula of HFUT YZS-16-01 is fully prepared (Fig. 3b, c). It has a typical shape of eosauropterygians, with a constriction separating a ventral glenoidal portion from a posteriorly directed dorsal wing. This is a synapomorphy of eosauropterygians. The dorsal wing of the scapula tapers to a blunt tip. The coracoid has a roughly rounded contour, and the coracoid foramen is open (see Fig. 3d, e). However, there is a narrow indentation in the posterolateral margin of the coracoid, an autapomorphy among sauropterygians. The pectoral fenestration is present in the holotype.

### Forelimb

**Fig. 4:**
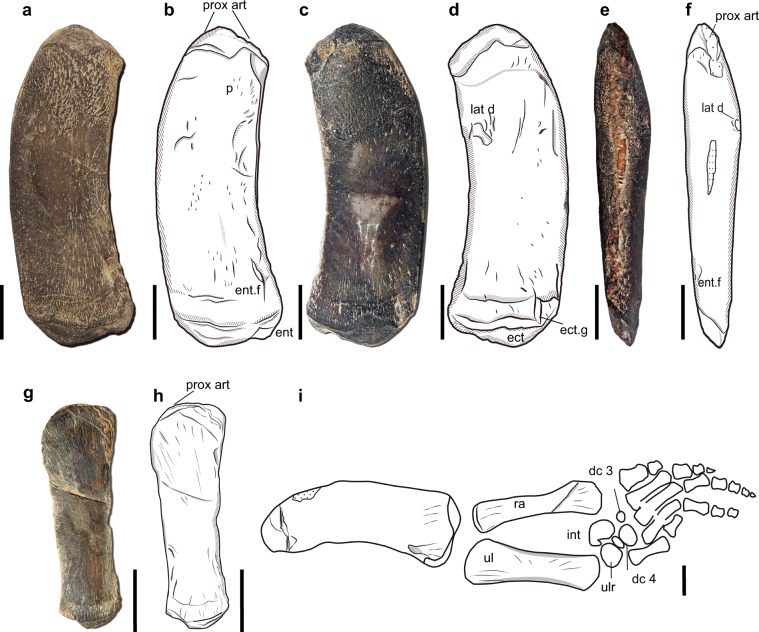
The forelimb of *Lariosaurus sanxiaensis*. **a**, **b** Photograph and drawing of right humerus of HFUT YZS-16-01 in ventral view. **c**, **d** Photograph and drawing of right humerus of HFUT YZS-16-01 in dorsal view. **e**, **f** Photograph and drawing of right humerus of HFUT YZS-16-01 in posterior view. **g**, **h** Photograph and drawing of ulna of HFUT YZS-16-01. **i** Drawing of the right forelimb of YIGM V 0940 in ventral view (Modified after Cheng^27^; Note the exchange of the position of radius and ulna compared with Cheng^27^). c carpal, dc distal carpal, lat d insertional crest for latissimus dorsi muscle, ect ectepicondyle, ect. g ectepicondylar groove, ent entepicondyle, ent. f entepicondyle foramen, p attachment of pectoralis muscle, prox art proximal screw-shaped area for articulation, ul ulna, ulr ulnare. Scale bars equal 2 cm.

In HFUT YZS-16-01, only the right humerus and ulnae are preserved (Fig. 4a–g). The humerus is still in its original position, but the ulna has been dislocated. The right humerus of HFUT YZS-16-01 was fully prepared (Fig. 4a–f). Dense striations are present all over the humerus. The humerus is evenly curved and its mid-shaft is broader than the proximal head. The humeral head is well developed, indicating an advanced ontogenetic stage of the individual. Both the deltopectoral crest and the insertional crest for latissimus dorsi muscle are much reduced (Fig. 4a–d). In posterior view, the crest for latissimus dorsi muscle of the humerus is much thicker than other parts of the bone (Fig. 4e, f). The epicondyle of humerus is only weakly developed. The ectepicondylar groove is deeply notched distally. The entepicondylar foramen is distinct in ventral view. The distal articular surface for the ulna is much wider than for the radius. Compared with the standard length^28^ of the specimen, the humerus is distinctly small, showing another autapomorphy of the species.

In the holotype, the left forelimb is relatively complete, except for the missing of some distal phalanges (Fig. 4h). The radius is approximately of equal length with the ulna. Compared with the radius, the proximal head and the middle shaft of the ulna are both distinctly expanded. The distal end of the ulna is as equally wide as the middle shaft in both specimens. Different from all other sauropterygians, the ulna in *Lariosaurus sanxiaensis* has quite straight preaxial and postaxial margins. The holotype preserves five carpal ossifications. It is clear that hyperphalangy is moderately developed in the manus of *L. sanxiaensis*, as evidenced by the preservation of five phalanges in digit 3 of the holotype (Fig. 4i).

### Phylogenetic analysis

An evenly curved humerus and a constricted scapula with a ventral glenoidal portion and a posteriorly directed dorsal wing make the identification of HFUT YZS-16-01 as an eosauropterygian unequivocal. In the Nanzhang-Yuan’an fauna, two eosauropterygians have been recognized: *Hanosaurus hupehensis*^29^ and *Lariosaurus sanxiaensis*. HFUT YZS-16-01 shares several apomorphies with the holotype of *L. sanxianensis*, including ventrally shifted transverse processes in the dorsal region, distinctly small humeri, and relatively straight ulna. *L. sanxiaensis* differs from *Hanosaurus* in retaining round coracoids. In addition, Cheng^27^ described some other differences in the pelvic girdle and hind limbs based on a referred specimen of *L. sanxiaensis* (YIGM V 0941), but he has never described or figured the referred specimen.

In order to assess the phylogenetic relationships of *Lariosaurus sanxiaensis* among eosauropterygians, we compiled a novel data matrix modified and expanded from Liu et al.^19^. Araeoscelidia, Younginiformes, Archosauromorpha and *Placodus* were selected as consecutive outgroups. Compared with Liu et al.^19^, several new taxa including *Hanosaurus*, *Yunguisaurus*, *Diandongosaurus*, *Majiashanosaurus* and *Bobosaurus* were added to the data matrix as ingroups^16,29–31^.

There are several other new eosauropterygians described after the publication of Liu et al.^19^. These include *Qianxisaurus*, *Odoiporosaurus*, *Dianmeisaurus*, *Dawazisaurus* and *Wangosaurus*^15,32–35^. *Qianxisaurus*, *Odoiporosaurus*, *Dianmeisaurus* and *Dawazisaurus* were not included in the data matrix because we have not yet examined the taxa personally. *Wangosaurus* was not included in the data matrix because the holotype has been further prepared and currently restudied by colleagues. For all other ingroups, at least one specimen for each taxon has been examined personally by one of us (JL). In addition, the four European pachypleurosaurian genera have been coded separately to test their monophyly. Species of *Nothosaurus* and *Lariosaurus* as traditionally recognized have also been coded separately because the genera are paraphyletic^11^. Only those species with the preservation of an articulated cranial and postcranial skeleton have been included in the current data matrix. Finally, we coded Plesiosauria based on *Rhaeticosaurus*, *Stratesaurus*, *Avalonnectes*, *Eurycleidus*, *Atychodracon*, *Thalassiodracon*, and *Eoplesiosaurus*.

Via the comparative study, 44 more or less new characters were added into the data matrix. This results in a new data matrix containing 36 taxa and 181 characters in total. The character description and the new data matrix consisting of 36 taxa and 181 characters are provided in Supplementary Notes and Supplementary Data 1, respectively. Personally examined specimens and referred literature are given in Supplementary Table 1.

**Fig. 5:**
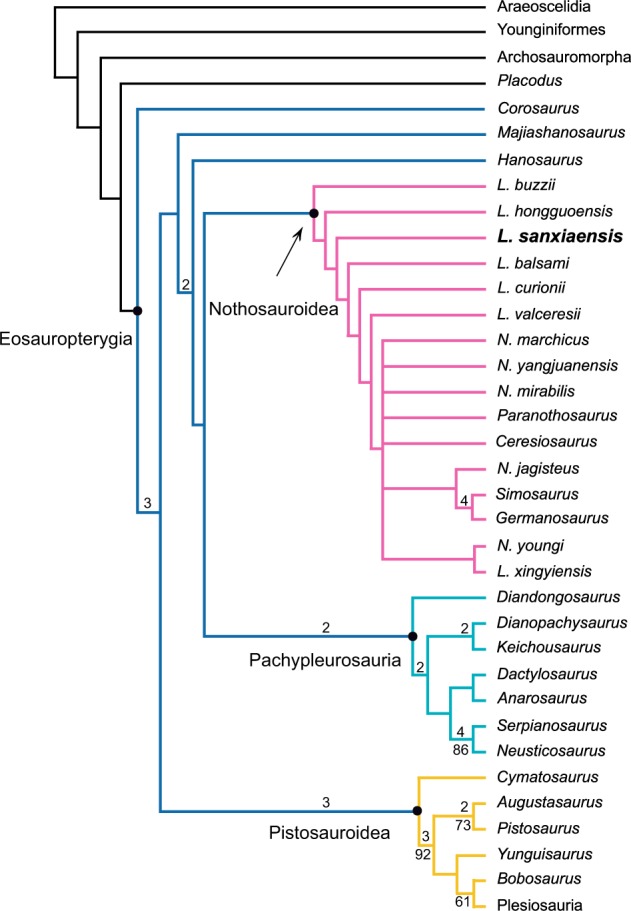
Strict consensus tree of five most parsimonious trees illustrating the phylogenetic position of *Lariosaurus sanxiaensis* inside eosauropterygians (TL = 618; CI = 0.356; RI = 0.623). Bootstrap values over 50% (with 1000 replicates) and Bremer support over 1 are indicated in the tree. *Nothosaurus* and *Lariosaurus* are abbreviated in the figure.

Our phylogenetic analysis was conducted in PAUP 4.0a 166 using the same settings of Liu et al.^19^. Five equally most parsimonious trees were obtained by heuristic search (Fig. 5). *Lariosaurus sanxiaensis* is recovered as a basal Nothosauroidea. Different from the traditional topology of Sauropterygia^14^, Pachypleurosauria comprises the sister group of Nothosauroidea. The Early Triassic *Hanosaurus* and *Majiashanosaurus* comprise the consecutive sister groups of the new clade including Pachypleurosauria and Nothosauroidea, while the Early Triassic *Corosaurus* becomes the sister group of all other eosauropterygians.

## Discussion

Several eosauropterygian phylogenies have been published recently, especially with the description of new taxa^19–21,35,36^, but these studies mostly relied on the original data matrix of Rieppel et al.^37^ and few characters have been modified. Rieppel et al.^37^ divided Eosauropterygia into Pachypleurosauria, Nothosauroidea and Pistosauroidea. This traditional topology has received support from several recent studies^19,20^. However, Holmes et al.^38^ challenged the monophyly of Pachypleurosauria, and many later studies lent support to the collapse of the monophyly of the clade^16,21–23,35,36^.

In this study, the data matrix from Liu et al.^19^ is greatly expanded up to 181 characters. Based on the new data matrix, the monophyly of traditionally recognized Pachypleurosauria^14^ is supported in our phylogenetic analysis as in some of previous studies^19,20^. This clade is diagnosed by several unambiguous characters including relatively smoothed bones of dermatocranium, relatively short snout, weakly developed snout constriction in the anterolateral margin of external naris, unretracted external nares, presence of quadratojugal, and absence of anterolaterally expanded corners of clavicles.

However, different from the traditional topology where Pachypleurosauria forms the sister group of Eusauropterygia, in our analysis Pachypleurosauria forms the sister group of Nothosauroidea. This topology is similar to a previous study^20^, but different from most other studies^19–23,35,36^. The Early Triassic pachypleurosaur-like forms *Hanosaurus* and *Majiashanosaurus* comprise the consecutive sister groups of the new clade including Pachypleurosauria and Nothosauroidea. The basal positions of *Hanosaurus* and *Majiashanosaurus* in our phylogenetic tree correspond well to their early appearance in the geological record.

Nothosauroidea classically consists of *Simosaurus*, *Germanosaurus*, *Nothosaurus* and *Lariosaurus*, while *Simosaurus* and *Germanosaurus* comprise the consecutive sister groups of the Nothosauridae including *Nothosaurus* and *Lariosaurus*^14^. Although the cladistic analysis performed here did not specifically aim to resolve the phylogeny of Nothosauroidea, the result again shows the collapse of monophyly of the classically recognized *Nothosaurus* and *Lariosaurus*, as in previous research^11,39^. Another unusual result of the analysis is the position of the clade consisting of *Germanosaurus* and *Simosaurus*, which is different from all of previous studies in that this clade is deeply nested in the Nothosauroidea. Despite these apparent differences, we noticed that the statistical support for the topology of Nothosauroidea present here is generally low (Fig. 5). Therefore, we refrain to revise the taxonomy of Nothosauroidea pending further comprehensive investigation of nothosaurs in the future.

**Fig. 6:**
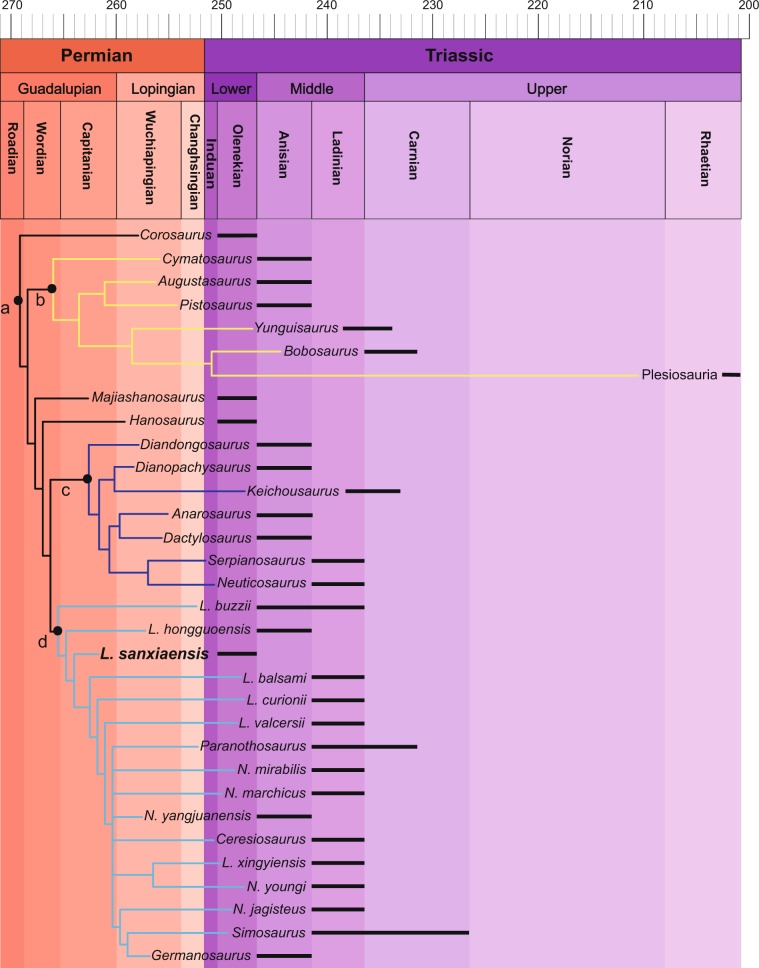
Phylogenetic relationship and stratigraphic occurrences of Eosauropterygia. *Nothosaurus* and *Lariosaurus* are abbreviated in the figure. **a** Eosauropterygia, **b** Pistosauroidea, **c** Pachypleurosauria, **d** Nothosauroidea.

Our result indicates the collapse of monophyly of Eusauropterygia as in most previous studies^16,20–23,35,36^. The Pistosauroidea now forms the sister group of the clade including *Majiashanosaurus*, *Hanosaurus*, Pachypleurosauria and Nothosauroidea. It is diagnosed by several unambiguous characters including strongly reduced or absent nasals, separated nasals by the nasal process of premaxillae, sagittal crest formed by parietal skull table, elongated and “scoop”-like mandibular symphysis, and platycoelous vertebrae. However, the Early Triassic *Corosaurus* that was traditionally recognized as a pistosaur^14^ now occupies the basal-most position of eosauropterygians. The phylogenetic relationship and stratigraphic occurrence of eosauropterygians (Fig. 6) here show that the basal position of three Early Triassic eosauropterygians *Corosaurus*, *Majiashanosaurus* and *Hanosaurus* match the stratigraphy quite well compared with previous studies^16,20–23,35^.

To quantitatively test the stratigraphic fit of our phylogenetic consensus tree, we performed stratigraphic congruence analysis by calculating MSM*, GER* and GER. The result indicates a significant fit of our strict consensus tree to the stratigraphic record (Table 2, Tree T-SC). A comparison with the most recent phylogenetic analysis of eosauropterygians^22^ indicates our eosauropterygian trees match the stratigraphic record much better. Both the five most parsimony trees (Trees MPT 1E-5E in Table 2) and the strict consensus tree (Tree MPT 1-5EC in Table 2) of the eosauropterygian clade derived from our novel data matrix show lower *p*. GER and *p*. MSM* than the strict consensus tree (Tree MPT 1-7EJ) of the eosauropterygian clade in Jiang et al.^22^, indicating better stratigraphic fit in our trees.

**Table 2:** Summary of the stratigraphic congruence analysis.

Tree	GER	MSM*	*est.p*. GER	*est.p*. MSM*
T-SC	0.9547	0.5333	0.0009	0.00005
MPT 1E	0.6269	0.4054	0.0046	0.3829
MPT 2E	0.6004	0.3889	0.0126	0.4488
MPT 3E	0.6004	0.3889	0.0119	0.4452
MPT 4E	0.6004	0.3889	0.0144	0.4574
MPT 5E	0.6004	0.3889	0.0116	0.4263
MPT 1-5EC	0.6004	0.3889	0.0110	0.4711
MPT 1-7EJ	0.3737	0.3600	0.2382	0.7813

*T-SC* strict consensus tree including all taxa from this study, *MPT 1E - MPT 5E* five most parsimonious trees including only eosauropterygian taxa from this study, *MPT 1-5EC* strict consensus tree including only eosauropterygian taxa from this study, *MPT 1-7EJ* strict consensus tree including only eosauropterygian taxa from Jiang et al.^22^.

**Fig. 7:**
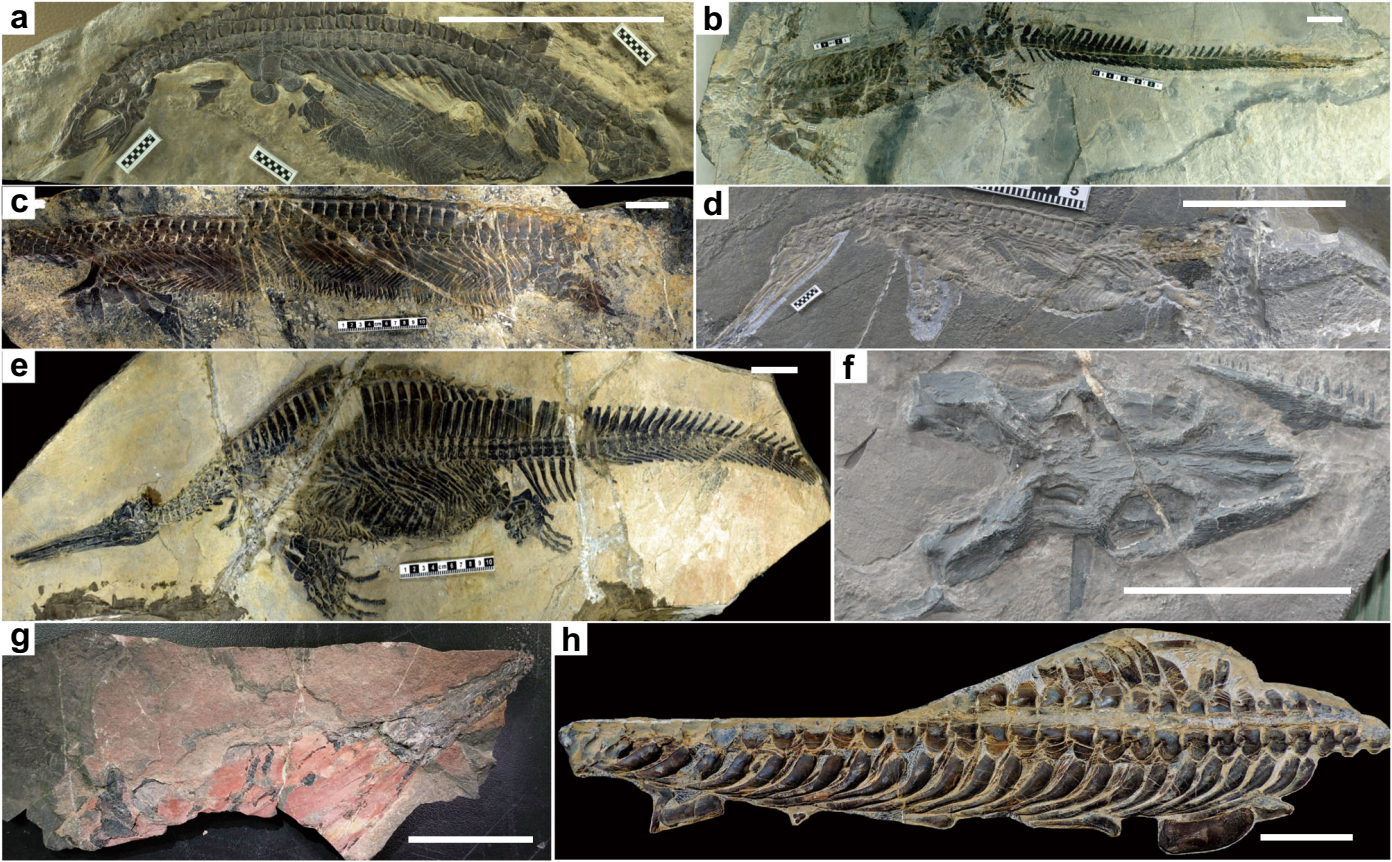
Diversified marine reptiles from Nanzhang-Yuan’an fauna. **a** *Nanchangosaurus suni* (WGSC 26006; modified after reference 44). **b** *Eretmorhipis carrolldongi* (WGSC V26020; modified after reference 46). **c** *Parahupehsuchus longus* (WGSC 26005; modified after reference 44). **d** *Eohupehsuchus brevicollis* (WGSC V26003; modified after reference 45). **e** *Hupehsuchus nanchangensis* (WGSC 26004; modified after reference 49). **f** *Hanosaurus hupehensis* (IVPP V3231). **g** *Chaohusaurus zhangjiawanensis* (HFUT YAV-10-08). **h** *Lariosaurus sanxiaensis* (HFUT YZS-16-01). Scale bars equal 5 cm.

The Nanzhang-Yuanan fauna where *Lariosaurus sanxiaensis* was discovered is an intriguing fauna. Since the first report of a Triassic marine reptile in the region^40^, the Nanzhang and Yuan’an counties have been extensively explored for Triassic marine reptile fossils, especially in the recent decade after the discovery of many new Triassic marine reptiles from South China^41^. Up to now, there are eight Triassic marine reptile taxa recognized from the Nanzhang-Yuan’an fauna (Fig. 7). These include the ichthyosaur *Chaohusaurus zhangjiawanensis*^42^, the hupehsuchians *Nanchangosaurus suni*^40^, *Hupehsuchus nanchangensis*^43^, *Parahupehsuchus longus*^44^, *Eohupehsuchus brevicollis*^45^ and *Eretmorhipis carrolldongi*^46^, and the sauropterygians *Hanosaurus hupehensis*^29^ and *L. sanxiaensis*^27^. Despite the detailed inspection in the last decades, neither fish nor invertebrate macrofossils have been found in the fauna^25^. Among these marine reptiles, most abundant are hupehsuchians, the size of which ranges from 40 cm (*Nanchangosaurus* and *Eohupehsuchus*) to approaching two meters (*Parahupehsuchus*), with *Hupehsuchus* and *Eretmorhipis* having an intermediate size of about one meter long. Hupehsuchians are a group of animals endemic to the Lower Triassic of Nanzhang-Yuan’an region. They have been hypothesized as a group of filter-feeding animals^47^, which limits them in feeding near the bottom of the trophic pyramid^48^ of Nanzhang-Yuan’an fauna, likely feeding on tiny crustaceans^25^ or other microorganisms. Their body was heavily built and well protected from predation^49^. The body tube present in many hupehsuchians was taken as evidence of marine tetrapod predation on other tetrapods^49^. This is further supported by the possible bite evidence left on the holotype of *Eohupehsuchus*^46^.

Although there are no tooth-bearing bones preserved in both specimens of *Lariosaurus sanxiaensis*, the predatory teeth present in all other nothosaurs^11^ suggest that such predatory teeth are likely present in *L. sanxiaensis*. The discovery of *L. sanxiaensis* provides a potential predator preying on hupehsuchians. To infer the size range of potential prey of *L. sanxiaensis*, it is necessary to know the size of the predator. However, both specimens of *L. sanxiaensis* preserve only parts of the skeleton. Fortunately, the standard length of HFUT YZS-16-01 can be measured directly.

**Fig. 8:**
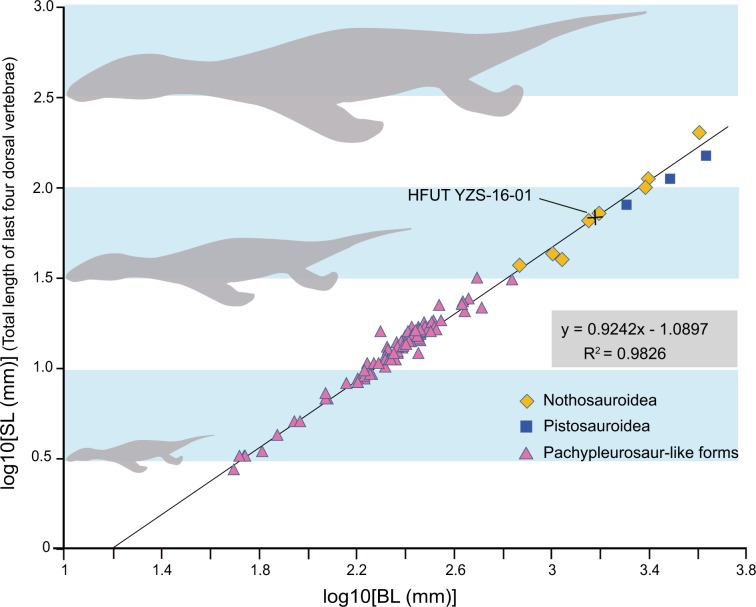
Standard length-body length relation in Triassic eosauropterygians. The black cross shows estimated length of *Lariosaurus sanxiaensis* (HFUT YZS-16-01).

The standard length was first proposed by Sander^28^ to estimate the body length of incomplete pachypleurosaur specimens. It was defined as the total length of the last four dorsal vertebrae. We compiled a database by recording the logarithms of standard length and the body length of all complete specimens of Triassic eosauropterygians (see Supplementary Table 2). Our result shows a close correlation between the two measurements among Triassic eosauropterygians (Fig. 8). The extrapolated body length of HFUT YZS-16-01 is about 1.5 m, which also supports the nothosaurian affinity of the species since pachypleurosaurs are generally smaller than this size, while pistosaurs are usually larger than this size^14^.

Such a medium-sized nothosaur would likely prey on other small marine reptiles including hupehsuchians present in this fauna. This inference is supported by the discovery of marine reptiles in the stomach contents of other nothosaurs^28,50^.

*Hanosaurus* is a small eosauropterygian. The inferred jaw adductor musculature^51^ suggests that *Hanosaurus* likely fed on small soft-bodied invertebrates, which were hard to be preserved as fossils. *Chaohusaurus* is a small basal ichthyosaur of which the adult body size is about one meter long. Its heterodont dentition and the body shape suggest that it may also feed on small invertebrates in the region^41^.

The inferred predator-prey relationships in the Nanzhang-Yuan’an fauna suggest a simple food web and shortened food chain in this fossil community. Compared with the middle Anisian Luoping biota from the same Yangtze carbonate platform^11,52^, the diversity and abundance of fish and macro-invertebrates in the community are much reduced, if not absent at all. Both faunae are well-preserved Lagerstätten in intraplatform basins of the same carbonate platform. Thus, the difference of fauna compositions was unlikely a preservation bias. Instead, it may reflect the true difference of ecosystems between Early and Middle Triassic in the shallow water platform of South China. Song et al.^8^ predicted shortened food chains in the Early Triassic fossil communities. The Nanzhang-Yuan’an fauna present here provides a direct line of evidence that food webs of Early Triassic fossil communities were simple and food chains were short. This supports the hypothesis that marine ecosystems in the Early Triassic were probably unhealthy and biotic recovery adopted a top-down pattern as suggested by Song et al.^8^, at least in the South China.

## Methods

### Preparation

The new specimen was originally exposed in dorsal view and the neural arches had been weathered away prior to the collection. Mounted needles and pneumatic tools were then used to prepare the specimen from the dorsal side. After the completion of the preparation of the dorsal side, the specimen was then mounted with silicone rubber and further prepared from the ventral side.

### Phylogenetic analysis

The character list (Supplementary Notes) and data matrix (Supplementary Data 1) were modified and updated from Liu et al.^19^ and constructed using NDE Version 0.5.0. Phylogenetic analysis was performed using the software PAUP Version 4.0a 166 for Windows^53^. Heuristic search (ADDSEQ = RANDOM, NREPS = 1000, HOLD = 100, with other settings default) was performed to acquire the most parsimonious trees.

### Stratigraphic congruence analysis

The modified Manhattan Stratigraphic Measure (MSM*)^54^ and the Gap Excess Ratio (GER)^55^ were calculated in this study. To calculate the metrics, the strict consensus tree and all of the most parsimony trees were time-calibrated. The StratPhyloCongruence function of Strap package^56,57^ in R^58^ was applied. Settings for stratigraphic congruence analysis are attached below: hard = F, randomly.sample.ages = T, samp.perm = 100, rand.perm = 100, fix.-topology = T, fix.outgroup = T. The R code for stratigraphic congruence analysis is based on Bell and Lloyd^56^. The first and last appearance data (FAD and LAD) datasets are available in Supplementary Tables 3 and 4.

### Reporting summary

Further information on research design is available in the Nature Research Reporting Summary linked to this article.

## Supplementary information

Supplementary Information

Description of Additional Supplementary Files

Supplementary Data 1

Reporting Summary

## Data Availability

All data generated or analysed during this study are included in this published article (and its supplementary information files). The fossil specimen is deposited at the paleontological laboratory of HFUT, with the accession number YZS-16-01.
