# Supplementary material for: An Early Triassic sauropterygian and associated fauna from South China provide insights into Triassic ecosystem health
Source: Commun Biol. 2020 Feb 11;3:63. doi: 10.1038/s42003-020-0778-7 (PMC7012838; doi:10.1038/s42003-020-0778-7)
Supplement: Supplementary file 1 — Supplementary Information [file 42003_2020_778_MOESM1_ESM.pdf]

Supplementary Notes

1. Character description

The characters are mainly cited and updated from Liu et al.[1], with several other characters from Rieppel[2] and Benson et al.[3]. The abbreviation of "L+number, R+number, and B+number" indicates the characters cited from Liu et al.[1], Rieppel[2] and Benson et al.[3] respectively.

[1] Bones in the dermatocranium (L1)

0, distinctly sculptured

1, relatively smooth

[2] Preorbital and postorbital region of skull (L2)

0, of subequal length

1, preorbital region distinctly longer

2, postorbital region distinctly longer

[3] Ratio, condylobasal skull length divided by longitudinal diameter of upper temporal fossa (modified from R16)

0, more than 3.8

1, 3.0-3.4

2, less than 2.9

[4] Ratio, the distance from the snout to the anterior margin of the internal naris divided by the distance from the snout to the anterior margin of the external naris (modified from R6)

0, smaller than 1.2

1, larger than 1.3

[5] Ratio, distance from posterior margin of external naris to anterior margin of orbit divided by the width of the postorbital arch (modified from R13)

0, more than 1.7

1, less than 1.5

[6] Snout (L3)

0, relatively short

1, elongated with broad anterior termination

2, elongated and tapering anteriorly

[7] Rostrum (modified from R1)

0, short

1, long and slender

[8] Snout constriction at the level of the anterolateral margin of the external naris in adult (modified from L4)

0, absent

1, strongly developed

2, weakly developed

[9] Premaxillae (L5)

0, small

1, large, forming most of snout in front of external nares

[10] Premaxilla(e) in adult

0, paired

1, partly or fully fused

[11] Postnarial process of premaxilla (L6)

0, absent

1, present, excluding maxilla from posterior margin of external naris

[12] Maxilla, depression at lateral margin of external naris and a foramen at its bottom for the exit of a lateral branch of the superior alveolar nerve (R 5)

0, absent

1, present

[13] External nares (L7)

0, not retracted

1, retracted with a longitudinal diameter approaching or exceeding half the longitudinal diameter of the orbit

2, retracted, narrow, and with a longitudinal diameter that is distinctly less than half the longitudinal diameter of the orbit

[14] Nasal(s), length (L8)

0, shorter than frontal(s)

1, longer than frontal(s)

[15] Nasal(s), size (L9)

0, not reduced

1, strongly reduced or absent

[16] Nasals in adult

0, paired

1, fused

[17] Nasal(s) (L10)

0, meet in dorsomedial suture

1, are separated from one another by nasal processes of the premaxillae extending back to the frontal bone(s)

[18] Nasal(s)

0, enter external naris

1, do not enter external naris

[19] Nasal, anterolateral process lining the entire medial margin of external naris (R 4)

0, absent

1, present

[20] Nasal, length behind level of posterior margin of external naris more than twice of the maximal width (R7)

0, absent

1, present

[21] Nasal, roughly triangular shape with a rectangular angle

0, absent

1, present

[22] Nasal-prefrontal contact

0, present

1, absent

[23] Lacrimal (L11)

0, is present and enters the external naris

1, remains excluded from the external naris

2, absent

[24] Dorsal exposure of prefrontal (L12)

0, large

1, reduced

[25] Prefrontal (L13)

0, without slender anteromedial process

1, with slender anteromedial process entering between maxilla and premaxilla

[26] Frontal (L14)

0, forms the dorsal margin of the orbit

1, excludes from the dorsal margin of the orbit by a contact of prefrontal and postfrontal

[27] Frontal(s) in adult (L15)

0, paired

1, fused

[28] Distinct posterolateral processes of frontal(s) (L16)

0, absent

1, present

[29] Frontal (L17)

0, widely separated from the upper temporal fossa

1, narrowly approaches the upper temporal fossa

2, enters the anteromedial margin of the upper temporal fossa

[30] Postfrontal (L18)

0, large and plate-like

1, with distinct lateral process overlapping the dorsal tip of the postorbital

2, with reduced lateral process and hence more of an elongate shape

[31] Postfrontal, distinct constriction behind the orbit

0, absent

1, present

[32] Postfrontal (R 10)

0, entering upper temporal fossa

1, excluded from upper temporal fossa

[33] Postorbital, forming all of the anterior margin of the upper temporal fossa (R12)

0, absent

1, present

[34] Jugal (modified from R14)

0, absent

1, present

[35] Jugal, anterior extension (modified from L19)

0, entering orbit

1, excluded from posterior margin of orbit

[36] Jugal, posterior extension (L20)

0, extends backwards no farther than to the middle of the cheek region

1, extends nearly to the posterior end of the skull

[37] Jugal (L21)

0, remains excluded from the upper temporal arch

1, enters the upper temporal arch

[38] Jugal-squamosal contact

0, absent

1, present

[39] Parietal(s) in adult (L22)

0, paired

1, fused in their posterior part only

2, fully fused

[40] Parietal skull table (L23)

0, broad

1, weakly constricted

2, strongly constricted (at least posteriorly)

3, forming a sagittal crest

[41] Parietal skull table, constriction in the posteriormost part

0, absent

1, present

[42] Pineal foramen (L24)

0, close to the middle of the skull table

1, weakly displaced posteriorly

2, strongly displaced posteriorly

3, displaced anteriorly

4, absent

[43] Pineal foramen located within a deep trough

0, absent

1, present

[44] Postparietals (L25)

0, present

1, absent

[45] Tabulars (L26)

0, present

1, absent

[46] Supratemporals (L27)

0, present

1, absent

[47] Temporal region of skull (L28)

0, relatively high

1, strongly depressed

[48] Upper temporal fossa (L29)

0, absent

1, present and subequal in size or slightly larger than the orbit

2, present and distinctly larger than orbit

3, present and distinctly smaller than orbit

[49] The anteromedial corner of the upper temporal fossa (L30)

0, is not or only slightly

1, is fully floored by a descensus from the postorbital, which together with neighboring elements (postfrontal, parietal) separates it from orbit

[50] Upper temporal fenestra, constriction of anterior corner (modified from R18)

0, absent

1, present

[51] Lower temporal fossa (L31)

0, absent

1, present and closed ventrally

2, present but open ventrally

[52] Squamosal (L32)

0, descends to ventral margin of skull

1, remains broadly separated from ventral margin of skull

[53] A box-like suspensorium of the squamosal (L33)

0, absent

1, present

[54] Distinct notch of squamosal to receive distal tip of paroccipital process (L34)

0, absent

1, present

[55] Quadratojugal (L35)

0, present

1, absent

[56] Anterior process of quadratojugal (L36)

0, present

1, absent

[57] Quadrate (L37)

0, covered by squamosal and quadratojugal in lateral view

1, exposed in lateral view

[58] Posterior margin of quadrate in lateral view (modified from L38)

0, straight

1, concave

[59] Lateral conch on quadrate in posterior view (modified from L39)

0, absent

1, present

[60] Dorsal wing of epipterygoid (L40)

0, approximately as broad as its base

1, narrower than its base

[61] Braincase (L41)

0, located at posterior end

1, deeply recessed below parietal skull roof (or parietal sagittal crest)

[62] Occipital crest (L42)

0, absent

1, present but squamosals do not meet behind parietal

2, present and squamosals meet behind parietal

[63] Occiput (L43)

0, with paroccipital process forming the lower margin of the posttemporal fossa and extending laterally

1, paroccipital processes trending posteriorly

2, plate-like with no distinct paroccipital process and with strongly reduced posttemporal fossae

[64] Mandibular articulations (L44)

0, approximately at level with occipital condyle

1, displaced to a level distinctly behind occipital condyle

2, positioned anterior to the occipital condyle

[65] Supraoccipital (L45)

0, exposed more or less vertically on occiput

1, exposed more or less horizontally at posterior end of parietal skull table

2, U-shaped

[66] Supraoccipital, sagittal crest

0, absent

1, reduced

2, prominent

3, knob-like

[67] Contact between exoccipitals above the basioccipital condyle (L46)

0, present

1, absent

[68] Basioccipital tubera (L47)

0, free

1, in complex relation to the pterygoid, as they extend ventrally

2, in complex relation to the pterygoid, as they extend laterally

[69] Palate (L48)

0, kinetic

1, akinetic

[70] Premaxillae (L49)

0, enter internal naris

1, are excluded from internal naris

[71] Posterior palatine vacuities (L50)

0, absent

1, present

[72] Pterygoids (L51)

0, longer than palatines

1, shorter than palatines

[73] Pterygoid flanges (L52)

0, well developed and transversely oriented

1, well developed and longitudinally oriented

2, strongly reduced

[74] Ectopterygoid (L53)

0, present

1, absent

[75] Suborbital fenestra (L54)

0, absent

1, present

[76] Internal carotid passage (L55)

0, enters basicranium

1, enters quadrate ramus of pterygoid

[77] Splenial bone (L56)

0, enters the mandibular symphysis

1, remains excluded therefrom

[78] Distinct coronoid process of lower jaw (L57)

0, absent

1, present

[79] Strongly projecting lateral ridge of surangular defining the insertion area for superficial adductor muscle fibers on the lateral surface of the lower jaw (L58)

0, absent

1, present

[80] Mandibular symphysis (L59)

0, short

1, elongated and "scoop"-like

[81] Mandibular symphysis, anterior fusion

0, absent

1, present

[82] Mandible, anterior constriction

0, absent

1, present

[83] Retroarticular process of lower jaw (L60)

0, absent

1, present

[84] Retroarticular process

0, short

1, long

[85] Trough on dorsal surface of retroarticular process (L61)

0, absent

1, present

[86] Retroarticular process dorsal surface, foramen for the innervation of chorda tympani nerve

0, absent

1, present

[87] Retroarticular process, trough on lateral surface for the insertion of the depressor mandibulae muscle/superficial pterygoideus muscle

0, absent

1, weak

2, strong

[88] Retroarticular process, trough on medial surface for the insertion of posterior fibers of pterygoideus internus muscle

0, absent

1, present

2, small distal notch present

[89] Teeth (L62)

0, set in shallow or deep sockets

1, superficially attached to bone

[90] Durophagous dentition, including much enlarged palatine tooth plates (L63)

0, absent

1, present

[91] Anterior (premaxillary and dentary) teeth (L65)

0, upright or only slightly procumbent

1, strongly procumbent

[92] Enlarged premaxillary and anterior dentary teeth (L66)

0, absent

1, present

[93] Premaxillary teeth (Modified after L64)

0, 4,

1, more than 5

2, 5

3, 3 or less

[94] Premaxillary fangs (modified from R2)

0, absent

1, present but less than 5

2, 5 or more

[95] One or two enlarged teeth on maxilla (L67)

0, present

1, absent

[96] The maxillary tooth row (L68)

0, is restricted to a level in front of the posterior margin of the orbit

1, extends backwards to a level below the posterior corner of the orbit and/or the anterior corner of the upper temporal fossa

2, extends backwards to a level below the anterior one third to one half of the upper temporal fossa

[97] Number of small maxillary teeth anterior to the maxillary fang(s)

0, 3 or less

1, 4,

2, 5 or more

[98] Dentary fangs

0, absent

1, present but less than 5

2, 5 or more

[99] Teeth on pterygoid flange (L69)

0, present

1, absent

[100] Vertebrae (L70)

0, notochordal

1, non-notochordal

[101] Vertebrae, shape (L71)

0, amphicoelous

1, platycoelous

2, or other

[102] Vertebral centrum (L72)

0, distinctly constricted in ventral view

1, with parallel lateral edges

[103] Subcentral foramina (L73)

0, absent

1, present

[104] Zygosphene-zygantrum articulation (L74)

0, absent

1, present

[105] Zygapophyseal pachyostosis (L75)

0, absent

1, present

[106] Number of cervical vertebrae (modified from L76)

0, less than 13,

1, 14-27

2, more than 28

[107] Cervical centra (L77)

0, rounded ventrally

1, keeled ventrally

[108] Parapophysis (L78)

0, does not shift backwards on centrum along the cervical vertebral column

1, does shift backwards on centrum along the cervical vertebral column

[109] Cervical intercentra (L79)

0, present

1, absent

[110] Cervical vertebrae, proportions of anterior cervical neural spines (modified from B131)

0, taller than their anteroposterior length

1, longer than tall

[111] Rib facets of the anterior-middle cervical vertebrae (modified from B122)

0, separated

1, co-jointed

[112] Neural spines on dorsal vertebrae (modified from R25)

0, low

1, tall

[113] Distal articular surface on transverse processes of dorsal vertebrae (L80)

0, oblong

1, evenly rounded

[114] Transverse processes of neural arches of the dorsal region (L81)

0, relatively short

1, distinctly elongated

[115] Distal end of transverse processes of dorsal vertebrae (L82)

0, not increasing in diameter

1, distinctly thickened

[116] Sutural facets receiving the pedicels of the neural arch on the dorsal surface of the centrum in the dorsal region (L83)

0, narrow

1, expanded into a cruciform or "butterfly-shaped" platform

[117] Dorsal intercentra (L84)

0, present

1, absent

[118] Anteroposterior trend of increasing inclination of pre- and postzygapophyses within the dorsal and sacral region (L85)

0, absent

1, present

[119] Elongation of neural spines in proximal tail region (R125)

0, absent

1, present

[120] A distinct free anterior process of cervical ribs (L86)

0, absent

1, present

[121] Pachyostosis of dorsal ribs (L87)

0, absent

1, present

[122] Distinct groove on the posterior aspect of the proximal shoulder region of the dorsal ribs

0, absent

1, present

[123] The number of sacral ribs (modified from L88)

0, two

1, three

2, four

3, five

[124] Distinct expansion of distal head of sacral ribs (L89)

0, present

1, absent

[125] Sacral (and caudal) ribs or transverse processes and their respective centrum (L90)

0, sutured

1, fused

[126] Mineralized sternum (L91)

0, absent

1, present

[127] Median gastral element (L92)

0, angulated

1, straight

[128] The medial gastral rib element (L93)

0, always only has a single lateral process

1, may have a two-pronged lateral process

2, with the lateral process contributing to the formation of the plastron

[129] Cleithrum (L94)

0, present

1, absent

[130] Clavicles, shape (L95)

0, broad medially

1, narrow medially

[131] Clavicles (L96)

0, do not meet in front of the interclavicle

1, meet in an interdigitating anteromedial suture

[132] Anterolaterally expanded corners of clavicles (L97)

0, absent

1, present

[133] Clavicle (L98)

0, applied to the anterior (lateral) surface of scapula

1, applied to the medial surface of scapula

[134] The relationship between clavicles and interclavicle (L99)

0, in simple overlapping contact

1, anteromedioventral end of clavicle embraces lateral tip of interclavicle in a complex contact

[135] Interclavicle (modified from L100)

0, rhomboidal

1, T-shaped

2, Triangular

[136] Posterior process on (T-shaped) interclavicle (L101)

0, elongate

1, short

2, rudimentary or absent

[137] Scapula (L102)

0, represented by a broad blade of bone

1, with a constriction separating a ventral glenoidal portion from a posteriorly directed dorsal wing

[138] The dorsal wing or process of the eosauropterygian scapula (L103)

0, tapers to a blunt tip

1, is ventrally expanded at its posterior end

[139] Supraglenoid buttress (L104)

0, present

1, absent

[140] Number of coracoid ossifications (L105)

0, one

1, two

[141] Coracoid in ventral view (modified from L106)

0, with roughly rounded contours

1, strongly waisted

2, with expanded medial symphysis

3, co-ossified with scapula

[142] Coracoid foramen (L107)

0, enclosed by coracoid ossification

1, between coracoid and scapula

[143] Pectoral fenestration (L108)

0, absent

1, present

[144] Limbs (L109)

0, short and stout

1, long and slender

[145] Humerus (L111)

0, rather straight

1, curved

[146] Humerus, preaxial margin of shaft in dorsal-ventral view

0, with straight angle

1, smoothly curved

2, straight

[147] Humerus, proximal width compared with the mid-shaft (R123)

0, greater

1, less

[148] Deltopectoral crest (L112)

0, well developed

1, reduced or absent

[149] Insertional crest for latissimus dorsi muscle (L113)

0, prominent

1, reduced

[150] Epicondyles of humerus (L114)

0, prominent

1, reduced

[151] Ectepicondylar groove (L115)

0, open and notched anteriorly

1, open without anterior notch

2, closed

3, absent

[152] Entepicondylar foramen (L116)

0, present

1, absent

[153] Radius (L117)

0, shorter than ulna

1, longer than ulna

2, approximately of the same length with ulna

[154] Ulna, distinctly broadened proximal head compared with the radius (modified from R121)

0, absent

1, present

[155] Ulna, middiaphysis (R120)

0, as slender as radius

1, distinctly broadened compared with radius

[156] Distal end of ulna (Modified after L118)

0, not expanded compared with the middle shaft

1, distinctly expanded compared with the middle shaft (Sander et al., 1997)

[157] Ulna, anterior margin

0, smooth

1, with a tuberosity

[158] Total number of carpal ossifications (L119)

0, more than 3,

1, 3,

2, 2

[159] Hyperphalangy in manus (R122)

0, absent

1, present

[160] Iliac blade (L120)

0, well developed

1, reduced but projecting beyond level of posterior margin of acetabular portion of ilium

2, reduced and no longer projecting beyond posterior margin of acetabular portion of ilium

3, absent, i.e. reduced to simple dorsal stub

[161] Acetabular portion of ilium

0, broadened

1, rod-like

[162] An elongated iliac shaft

0, absent

1, present

[163] Spina praeacetabuli

0, absent

1, weakly developed

2, well developed

[164] Pubis (L121)

0, with convex ventral (medial) margin

1, with concave ventral (medial) margin

[165] Obturator foramen in adult (L122)

0, closed

1, open

2, forming the thyroid fenestra

[166] Thyroid fenestra (L123)

0, absent

1, present

[167] Acetabulum (L124)

0, oval

1, circular

[168] Foot (L110)

0, short and broad

1, long and slender

[169] Femoral shaft (L125)

0, stout and straight

1, slender and sigmoidally curved

[170] Internal trochanter (L126)

0, well developed

1, reduced

[171] Intertrochanteric fossa (L127)

0, deep

1, distinct but reduced

2, rudimentary or absent

[172] Distal femoral condyles (L128)

0, prominent

1, not projecting markedly beyond shaft

[173] Anterior femoral condyle relative to posterior condyle (L129)

0, larger and extending further distally

1, smaller/equisized and of subequal extent distally

[174] Total number of tarsal ossifications (L130)

0, four or more

1, three

2, two

[175] Perforating artery (L131)

0, passes between astragalus and calcaneum

1, between the distal heads of tibia and fibula proximal to the astragalus

[176] The proximal concavity of astragalus (L132)

0, absent

1, present

[177] Calcaneal tuber (L133)

0, absent

1, present

[178] Distal tarsal 1 (L134)

0, present

1, absent

[179] Distal tarsal 5 (L135)

0, present

1, absent

[180] Metatarsal 5, length (L136)

0, long and slender

1, distinctly shorter than the other metatarsals and with a broad base

[181] Metatarsal 5 (L137)

0, straight

1, "hooked"

2. Notes on character modifications

When the character coding is different from Liu et al.[1] and several others, the modification and the reason (where possible) are given below.

CHARACTER 2: *Placodus* is recoded as 2. *Augustasaurus* is recoded as 1 (see Rieppel et al.[4], p.581, Fig. 3A). *Hanosaurus* is recoded as 2.

CHARACTER 8: An additional state of a weakly snout constriction in adult is added, which is present in *Keichousaurus* (noted by Rieppel and Lin[5]), *Augustasaurus*[4], and also in *Dianopachysaurus* (LPV 31365)[1]. A weakly developed snout constriction is also developed in some juvenile specimens of *Simosaurus* and a few *Lariosaurus* specimens (noted by Rieppel[6]). However, they are still coded as 0. The preservation of the holotype of *Hanosaurus* cannot allow the unequivocal decision for this character.

CHARACTER 13: *Corosaurus* is recoded as 2.

CHARACTER 14: *Augustasaurus* and *Pistosaurus* are coded as ? where the presence/absence of nasals cannot be unequivocally determined.

CHARACTER 15: *Cymatosaurus* is recoded as 1, as noticed by Cheng et al.[7]. Preservation of *Pistosaurus* and *Augustasaurus* is not good enough to decide the coding of this character and they are coded as either reduced or absent. *Simosaurus* is recoded as 0 (see Rieppel[8], p.59, Fig. 47A).

CHARACTER 26: *Cyamodus* is recoded as 0.

CHARACTER 28: *Pistosaurus* is recoded from 1 to 0.

CHARACTER 39: *Yunguisaurus* was originally coded as 0 by Chen et al.[7]. Paired parietals were also confirmed by Zhao et al.[9]. Most recently Sato et al.[10] recoded this character as unknown for *Yunguisaurus*, but they indicated that the parietals are paired and the suture separate the parietals are visible posterior to the pineal foramen, which is located at the fronto-parietal boundary. We code *Yunguisaurus* here as 0 as in Chen et al.[7] based on personal observation. *Pistosaurus* is recoded from 1 to 0.

CHARACTER 44: *Yunguisaurus* is recoded from ? to 1.

CHARACTER 45: *Yunguisaurus* is recoded from ? to 1.

CHARACTER 46: *Yunguisaurus* is recoded from ? to 1.

CHARACTER 47: *Simosaurus* is recoded as 1.

CHARACTER 55: *Placodus* is recoded as 1 here because an absence of quadratojugal in other sauropterygians is also probably caused by the fusion of quadratojugal and squamosal. We can never decide if the absence of a quadratojugal is caused by the fusion of the quadratojugal and squamosal or the truncated development unless an ontogenetic sequence is available.

CHARACTER 56: *Placodus* is recoded as ?.

CHARACTER 59: *Yunguisaurus* is recoded from ? to 0.

CHARACTER 60: *Yunguisaurus* is recoded from ? to 1.

CHARACTER 61: *Yunguisaurus* is recoded from ? to 1.

CHARACTER 62: The occipital crest is absent in *Keichousaurus* based on personal observation. *Simosaurus* is recoded from 0 to 1. *Yunguisaurus* is recoded from ? to 2.

CHARACTER 65: *Germanosaurus* is recoded from ? to 0.

CHARACTER 76: *Yunguisaurus* is recoded from 0 to 1.

CHARACTER 78: *Dianopachysaurus* is recoded as 1.

CHARACTER 79: *Yunguisaurus* is recoded from 0 to 1.'

CHARACTER 80: *Keichousaurus* is recoded as 0. *Yunguisaurus* is recoded as 2.

CHARACTER 92: The premaxillary and anterior dentary fangs are clear present in *Yunguisaurus*. *Dianopachysaurus* and *Keichousaurus* are recoded as 1.

CHARACTER 95: Caniniform teeth are present in *Yunguisaurus* as pointed out by Zhao et al.[9]. *Anarosaurus* is recoded from 1 to 0 based on Klein [11].

CHARACTER 98: *Anarosaurus* is recoded from ? to 1+2 based on Klein [11].

CHARACTER 103: *Yunguisaurus* is recoded as 1.

CHARACTER 107: *Yunguisaurus* is recoded as 1.

CHARACTER 113: *Yunguisaurus* and *Bobosaurus* are recoded as 0.

CHARACTER 114: *Bobosaurus* is recoded from ? to 0.

CHARACTER 121: *Keichousaurus* is recoded from 1 to 0+1; *Bobosaurus* is

recoded from ? to 0.

CHARACTER 126: *Bobosaurus* is recoded from ? to 0.

CHARACTER 130: *Dianopachysaurus* is recoded as ?.

CHARACTER 136: *Anarosaurus* is recoded from ? to 2 based on Klein[12]. *Yunguisaurus* is recoded from ? to 2 based on Shang et al.[13].

CHARACTER 153: *Anarosaurus* is recoded from ? to 2 based on Klein[12].

CHARACTER 156: The short ulna of plesiosaurs with a convex postaxial margin is here treated as inapplicable; *Anarosaurus* is recoded from ? to 0 based on Klein[12].

CHARACTER 158: The holotype of *Yunguisaurus liae* is undoubtedly a juvenile specimen compared with ZMNH M8738 described by Zhao et al.[9]. There are apparently more than three carpal ossifications in the adult specimens of *Yunguisaurus* as present in ZMNH M8738; *Diandongosaurus* is recoded from 2 to 1+2.

CHARACTER 160: The sacral head of the ilium of *Hanosaurus* has reduced to simple stub. Different from Cheng et al.[7], *Yunguisaurus* is coded as 2 here. *Anarosaurus* is recoded from ? to 2 based on Klein[12].

CHARACTER 165: Obturator foramen in *Bobosaurus* is also absent as in *Yunguisaurus*.

CHARACTER 174: *Anarosaurus* is recoded from ? to 0 based on Klein[12].

CHARACTER 175: *Anarosaurus* is recoded from ? to 1 based on Klein[12].

CHARACTER 176: *Anarosaurus* is recoded from ? to 0 based on Klein[12].

CHARACTER 177: *Anarosaurus* is recoded from ? to 0 based on Klein[12].

CHARACTER 178: *Anarosaurus* is recoded from ? to 1 based on Klein[12].

CHARACTER 179: *Majiashanosaurus* is recoded from ? to 1. *Anarosaurus* is recoded from ? to 1 based on Klein[12].

CHARACTER 180: *Anarosaurus* is recoded from ? to 0 based on Klein[12].

CHARACTER 181: *Anarosaurus* is recoded from ? to 0 based on Klein[12].

Supplementary Table 1. List of specimens examined personally by J.L. and literature referred to. *Nothosaurus* and *Lariosaurus* are abbreviated.

Institutional abbreviations as below:

AGM	Anhui Geological Museum, Hefei, Anhui, China.
BGM	Bath Geological Museum, Bath, England.
BGR	Bundesanstalt fur Geowissenschaften und Rohstoffe, Berlin, Germany.
BSP	Bayerische Staatssammlung für Paläontologie und Historische Geologie, Munich, Germany.
FMNH	Field Museum of Natural History, Chicago, USA.
GMPKU	Geological Museum of Peking University, Beijing, China.
Gö	Institut und Museum für Geologie und Paläontologie, Georg-August-Universität, Göttingen, Germany.
GPIT	Geologisch-Paläontologisches Institut, Univerität Tübingen, Germany.
HFUT	Paleontological lab of Hefei University, Hefei, Anhui, China.
IVPP	Institute for Vertebrate Paleontology and Paleoanthropology, Beijing, China.
CCCGS	Chengdu Center of China Geological Survey, Chengdu, China.
LWL	Museum für Naturkunde, Münster, Germany.
MB. R	Natural History Museum (Fossil Reptile Collection), Humboldt University, Berlin, Germany.
MCSNIO	Museo Civico di Scienze Naturali, Induno Olona, Italy.
MCSNM	Museo Civico di Storia Naturale, Milano, Italy.
MFSN	Museo Friulano di Storia Naturale, Udine, Italy.
MGSB	Museo y Laboratorio de Geologia, Seminario de Barcelona, Spain.
MNHN	Muséum National d'Histoire Naturelle, Paris, France.
NMNS	National Museum of Natural Science, Taiwan, China.
PIMUZ	Paläontologisches Institut und Museum der Universität Zürich, Zürich, Switzerland.
SMF	Forschungsinstitut und Naturmuseum Senckenberg, Frankfurt a.M., Germany.
SMNS	Staatliches Museum für Naturkunde, Stuttgart, Germany.
UMO	Urwelt-Museum Oberfranken, Bayreuth, Germany.
UW	University of Wyoming Museum of Natural History, Laramie, Wyoming，USA.
XNGM	Xingyi National Geopark Museum, Xingyi, Guizhou, China.
ZMNH	Zhejiang Museum of Natural History, Hangzhou, Zhejiang, China.

Taxa	Personally examined specimens	Referred literature
Trilophosaurus	FMNH PR259 (cast)	Gregory, 1945[14]<br>Demar and Bolt, 1981[15]<br>Heckert et al., 2001[16]<br>Spielmann et al., 2005[17]<br>Heckert et al., 2006[18]<br>Mueller and Parker, 2006[19]<br>Spielmann et al., 2007[20]<br>Nesbitt et al., 2015[21]
Macrocnemus	PIMUZ T 1559<br>PIMUZ T 2470<br>PIMUZ T 2472<br>PIMUZ T 2473<br>PIMUZ T 2474<br>PIMUZ T 2475<br>PIMUZ T 2476<br>PIMUZ T 2816<br>PIMUZ T 5918<br>PIMUZ T 2477<br>PIMUZ T 4822<br>GMPKU-P-3001<br>IVPP V 15001	Peyer, 1937[22]<br>Kuhn-Schnyder, 1962[23]<br>Rieppel and Gronowske, 1981[24]<br>Rieppel, 1989[25]<br>Li et al., 2007[26]<br>Zhang et al., 2010[27]<br>Jiang et al., 2011[28]<br>Fraser and Furrer, 2013[29]<br>Pritchard et al., 2015[30]
Langobardisaurus	MCSNB 2883<br>MCSNB 4860<br>MFSN 1921	Renesto, 1994[31]<br>Renesto and Dalla Vecchia, 2000[32]<br>Renesto et al., 2002[33]<br>Saller et al., 2013[34]<br>Pritchard et al., 2015[30]
Tanystropheus	MCSNM BES SC 111<br>MCSNM BES SC 265<br>MCSNM BES SC 1018<br>MCSNM V3663<br>MCSNM V3730<br>MFSN 25761<br>MFSN 26829<br>MFSN 31573<br>PIMUZ T 2482<br>PIMUZ T 2483<br>PIMUZ T 2484<br>PIMUZ T 2790<br>PIMUZ T 1277<br>PIMUZ T 2791<br>GMPKU-P-1527	Peyer, 1931[35]<br>Wild, 1973[36]<br>Wild, 1980a[37]<br>Wild, 1980[38]<br>Wild and Oosterink, 1984[39]<br>Dalla Vecchia, 2000[40]<br>Rieppel, 2001[2]<br>Renesto, 2005[41]<br>dalla Vecchia, 2006[42]<br>Li, 2007[43]<br>Nosotti, 2007[44]<br>Rieppel et al., 2010[45]<br>Pritchard et al., 2015[30]

	IVPP V 14472	
Dinocephalosaurus	IVPP V13767<br>IVPP V13898<br>LPV 30280<br>LPV 30174	Li, 2003[46]<br>Li et al., 2004[47]<br>Rieppel et al., 2008[48]<br>Liu et al., 2017[49]
Protorosaurus	NHMW 1943I4<br>NHMW 1974-1635 (cast)	Seeley, 1887[50]<br>Romer, 1947[51]<br>Evans and King, 1993[52]<br>Gottmann-Quesada and Sander, 2009[53]
Jesairosaurus	MNHN ZAR 6<br>MNHN ZAR 15	Jalil, 1997[54]
Placodus	UMO uncatologued<br>UMO BT 13<br>UMO BT 725<br>UMO BT 742<br>UMO BT 745<br>UMO BT 746<br>UMO BT 747<br>UMO BT 1210<br>UMO BT 2174<br>BGR 5578<br>SMF R.359<br>SMF R.360<br>SMF R.368<br>SMF R.4110<br>SMF R.4112<br>GMPKU-P-1054<br>BSP 1912 I 75<br>SMNS 51982<br>SMNS 54547<br>SMNS 54552<br>SMNS 54571<br>SMNS 59824<br>SMNS 59826<br>SMNS 91424	Rieppel, 1995[55]<br>Meyer, 1863[56]<br>Broili, 1912[57]<br>Rieppel, 2000[58]<br>Jiang et al., 2008[59]<br>Diedrich, 2010[60]
Corosaurus	FMNH PR 135<br>FMNH PR 243<br>FMNH PR 244<br>FMNH PR 480<br>FMNH PR 1382<br>UW 5485 cast in MCSNM	Case, 1936[61]<br>Kuhn, 1964[62]<br>Rieppel,1998[63]

Cymatosaurus	BGR X612<br>BGR X613<br>BGR X614<br>BGR X1489<br>BGR X1490<br>BGR X1491<br>BGR X9218<br>BGR X9882<br>BGR X9883<br>SMNS 10109<br>SMNS 10977	Schrammen, 1899[64]<br>Huene, 1944[65]<br>Rieppel, 1997[66]<br>Maisch, 2014[67]
Bobosaurus	MFSN 27285	Dalla Vecchia, 2006[68]<br>Fabbri et al., 2014[69]
Yunguisaurus	ZMNH M 8738	Sato et al., 2010[70]<br>Sato et al, 2013[71]<br>Shang et al., 2017[13]
Augustasaurus	FMNH PR 1974	Sander et al., 1997[72]<br>Rieppel et al., 2002[73]
Pistosaurus	UMO uncataloged<br>SMF R4041<br>SMNS 1932<br>SMNS 56984<br>SMNS 90076<br>SMNS 90765<br>GPIT 1753 (cast)	Meyer, 1839[74]<br>Meyer, 1847-55[75]<br>Sues, 1987[76]
Simosaurus	MB.R 52<br>FMNH 405 (cast of UC 405)<br>MNHN AC 9025<br>MNHN AC 9028<br>SMNS 7861<br>SMNS 7862<br>SMNS 7956<br>SMNS 10360<br>SMNS 11364<br>SMNS 14733<br>SMNS 15012<br>SMNS 16363<br>SMNS 16700<br>SMNS 17590<br>SMNS 18220<br>GPIT VIII & IX<br>GPIT NC03K07<br>MFSN 31870	Meyer, 1847-55 [75]<br>Huene, 1921[77]<br>Rieppel, 1994[6]<br>Rieppel, 1994[78]<br>Rieppel, 2000[79]<br>Dalla Vecchia, 2008[80]

	MFSN 34884<br>MFSN 34904<br>MFSN 34905<br>MFSN 34913	
Germanosaurus	NHMW uncatalogued	Gürich, 1891[81]<br>Koken, 1893[82]<br>Nopcsa, 1928[83]<br>Rieppel, 1997[66]
Ceresiosaurus	PIMUZ T2460 (cast)<br>PIMUZ T 2461<br>PIMUZ T 5151<br>PIMUZ T 5559	Peyer, 1931[84]<br>Hänni, 2004[85]<br>Quesada and Aguera, 2005[86]
L. balsami	PIMUZ T 4829 (cast)<br>PIMUZ T 4849 (cast)<br>PIMUZ T 4850 (cast)<br>PIMUZ T 4853 (cast)<br>PIMUZ T 4854 (cast)<br>PIMUZ T 4856 (cast)<br>SMF R. 13<br>MGSB M-502<br>MGSB M-506<br>MGSB M-509	Curioni, 1847[87]<br>Boulenger, 1896[88]<br>Bassani,1886[89]<br>Mariani, 1923[90]<br>Zapfe and König, 1980[91]<br>Ticli, 1984[92]<br>Mazin, 1985[93]<br>Kuhn-Schnyder, 1987[94]<br>Renesto, 1993[95]<br>Rieppel, 1994[96]
L. buzzii	PIMUZ T 2804	Tschanz, 1989[97]<br>Rieppel, 1998[98]
L. curionii	PHVR 1	Kuhn-Schnyder, 1987[94]<br>Rieppel, 1998[98]
L. hongguoensis	GMPKU-P-1011	Jiang et al., 2006[99]
L. valceresii	MCSNIO P701	Tintori and Renesto, 1990[100]<br>Renesto, 2004[101]
L. xingyiensis	IVPP V 11866<br>XNGM WS-30-R19	Li and Rieppel, 2002[102]<br>Lin et al., 2017[103]
N. marchicus	MB.R.1<br>MB.R.4<br>MB.R.5<br>MB.R.6<br>MB.R.27<br>SMF R-4572<br>SMNS 80460	Koken, 1893[82]<br>Edinger, 1921[104]<br>Rieppel and Wild, 1996[105]
N. venustus (N. marchicus)	SMF R4537	Koken, 1893[82]
N. schroederi (N. marchicus)	BGR X611<br>BGR X9790	Huene, 1944[65]

	BGR X9892	
N. mirabilis	BSP 1935 I 16	Münster, 1834[106]
	BSP 1952 XV 108	Meyer, 1847-55[75]
	SMF R473	Quenstedt, 1852[107]
	SMNS 7214	Berckhemer, 1930[108]
	SMNS 10806	Rieppel, 1993[109]
	SMNS 16433	Rieppel and Wild, 1996[105]
	SMNS 16851	
	SMNS 17213	
	SMNS 17822	
	SMNS 18001	
	SMNS 18058	
	SMNS 18210	
	SMNS 18475	
	SMNS 51972	
	SMNS 55298	
	SMNS 56685	
	SMNS 56286	
	SMNS 57047	
	SMNS 58815	
	SMNS 59074	
	SMNS 80204	
	SMNS 81311	
	SMNS 81655	
	SMNS 82025	
	SMNS 84575	
	SMNS 91468	
	UMO 1000	
	UMO BT667	
	UMO BT669	
	UMO BT671	
	UMO BT690	
	UMO BT724	
N. bergeri (N. mirabilis)	Gö 756-1	Meyer, 1847-55[75]
N. jagisteus	SMNS 56618	Rieppel, 2001[110]
Paranothosaurus	MFSN 15329	Peyer, 1939[111]
	MFSN 16849	Kuhn-Schnyder, 1966[112]
	MFSN 16850	
	MFSN 16851	
	MFSN 19288	
	MFSN 19866	
	PIMUZ T 4829	
	SMNS 55298	
N. yangjuanensis	GMPKU-P-1080	Jiang et al., 2006[113]

	GMPKU-P-3014<br>IVPP V 14294<br>IVPP V 14301	Shang et al., 2006[114]<br>Yin et al., 2014[115]
N. youngi	IVPP V 13590<br>XNGM WS-30-R19	Li et al., 2004[116]<br>Ji et al., 2014[117]
Anarosaurus	Gö Orig. Nr. 409-1<br>(cast)	Dames, 1890[118]<br>Rieppel, 1995[119]<br>Klein, 2009[11] ;<br>Klein, 2012[12]
Dactylosaurus	BGR X10000<br>SMNF R-4097 (cast)<br>FMNH PR2660 (cast)	Gürich, 1884[120]<br>Nopcsa, 1928[83]<br>Sues and Carroll, 1985[121]<br>Rieppel, 1993[122]
Neusticosaurus pusillus	FMNH PR 421<br>FMNH PR 2020<br>FMNH PR 2657	Nopcsa, 1928[83]<br>Sues and Carroll, 1985[121]<br>Rieppel, 1993[122]<br>Curioni, 1847[87]<br>Seeley, 1882[123]<br>Nopcsa, 1928[83]<br>Pinna, 1967[124]<br>Carroll and Gaskill, 1985[125]<br>Sander, 1989[126]<br>Rieppel and Lin, 1995[5]<br>Renesto, 2006[127]
Serpianosaurus	MCSNM BES SC 1017<br>MCSNM BES SC 1280<br>PIMUZ T 90<br>PIMUZ T 1071<br>PIMUZ T 3675<br>PIMUZ T 3931	Rieppel, 1989[128]<br>Rieppel and Lin, 1995[5]<br>Rieppel and Hagdorn,1997[129]<br>Hugi and Scheyer, 2012[130]<br>Beardmore et al., 2012[131]
Keichousaurus	GMPKU-P-1962<br>GMPKU-P-1154<br>HFUT JYS 15-001<br>HFUT JYS 15-002<br>HFUT JYS 15-003<br>HFUT JYS 15-004<br>HFUT JYS 15-005<br>HFUT JYS 15-006<br>HFUT JYS 15-007<br>HFUT JYS 15-008<br>HFUT JYS 15-009<br>HFUT JYS 15-010<br>HFUT JYS 15-011	Young, 1958[132]<br>Lin and Rieppel, 1998[133]<br>Holmes et al., 2008[134]<br>Cheng et al., 2009[135]<br>Motani et al., 2015[136]<br>Xue et al., 2015[137]

	HFUT JYS 15-012<br>HFUT JYS 15-013<br>HFUT JYS 15-014<br>HFUT JYS 15-015<br>HFUT JYS 15-016<br>HFUT JYS 15-017<br>HFUT JYS 15-018<br>HFUT JYS 15-019<br>HFUT JYS 15-020<br>HFUT JYS 16-001<br>HFUT JYS 16-002<br>HFUT JYS 16-003<br>HFUT JYS 16-004<br>HFUT JYS 16-005<br>SMF R 4941<br>SMF R 4944<br>XNGM WS-31-R9<br>XNGM WS-31-R10<br>XNGM WS-31-R11<br>XNGM WS-31-R18<br>XNGM WS-31-R41<br>BSP 1995 I 42	
Dianopachysaurus	LPV 31365	Liu et al., 2011[1]
Diandongosaurus	IVPP V17661<br>PKU<br>NMNS-000933-F0349<br>8	Shang et al., 2011[138]<br>Sato et al., 2014[139]<br>Liu et al., 2015[140]
Hanosaurus	IVPP V 3231	Young, 1972[141]<br>Rieppel, 1998[142]
Majiashanosaurus	AGM-AGB5954	Jiang et al., 2014[143]
Rhaeticosaurus	LWL-MFN P 64047	Wintrich et al., 2017[144]

Supplementary Table 2. List of all complete specimens of Triassic eosauropterygians with the measurements of total length (TL) and standard length (SL). *Nothosaurus* and *Lariosaurus* are abbreviated.

**Taxon**	**TL (mm)**	**SL (mm)**	**lg TL**	**lg SL**	**Specimen Number**	**Refered Literature**
Ceresiosaurus	1010	43	3.0043	1.6335	PIMUZ T2460	Peyer, 1931[84]
	2470	111	3.3927	2.0453	PIMUZ T4836	Peyer, 1931[84]
L. valceresii	1100	40	3.0414	1.6021	MSCNIO P 500	Tintori and Renesto, 1990[100]
L. xingyiensis	1400	67	3.1461	1.8261	V 11866	Li and Rieppel, 2002[102]
	740	37	2.8692	1.5682	XNGM WS-30-R-19	Lin et al., 2017[103]
Paranothosaurus	4010	200	3.6031	2.3010	PIMUZ T4829	Peyer, 1939[111]
N. yangjuanensis	2400	100	3.3802	2.0000	GMPKU-P-3014	Yin et al., 2014[115]
N. youngi	1550	72	3.1903	1.8573	WS-30-R24	Ji et al., 2014[117]
Serpianosaurus	680	32	2.8325	1.5051	PIMUZ T 3931	Rieppel, 1989[128]
	510	22	2.7076	1.3424	PIMUZ T 3675	Personal observation
Dactylosaurus	260	15	2.4150	1.1761	SMF R 4097a	Sues and Carroll, 1985[121]
Neusticosaurus	288	14.2	2.4594	1.1523	PIMUZ T 3671	Sander, 1989[126]
	330	16.7	2.5185	1.2227	PIMUZ T 3934	
	210	13	2.3222	1.1139	PIMUZ T 3615	
	450	25	2.6532	1.3979	PIMUZ T 3430	
	310	17	2.4914	1.2304	PIMUZ T 3932	

	170	8.5	2.2304	0.9294	PIMUZ T 3393	
	430	24	2.6335	1.3802	PIMUZ T 3445	
	490	33	2.6902	1.5185	MSNM V 3549	Carroll and Gaskill, 1985[125]
Keichousaurus	49.5	2.5	1.6946	0.3979	NMNS-chw-01	Cheng et al., 2009[135]
	74.5	4	1.8722	0.6021	NMNS-chw-02	
	55	3	1.7404	0.4771	NMNS-chw-03	
	55.2	3	1.7419	0.4771	NMNS-chw-04	
	297	18.2	2.4728	1.2601	NMNS-cyn 2002-01	
	241	13	2.3820	1.1139	NMNS-cyn 2002-02	
	212.5	12.5	2.3274	1.0969	NMNS-cyn 2002-03	
	286	15.5	2.4564	1.1903	NMNS-cyn 2002-04	
	216	11	2.3345	1.0414	NMNS-cyn 2002-05	
	325	18.5	2.5119	1.2672	NMNS-cyn 2002-06	
	296	16	2.4713	1.2041	NMNS-cyn 2002-07	
	283.8	14.5	2.4530	1.1614	NMNS-cyn 2002-08	
	230	13.8	2.3617	1.1399	NMNS-cyn 2002-10	
	248.5	15	2.3953	1.1761	NMNS-cyn 2002-11	
	300	17	2.4771	1.2304	NMNS-cyn 2002-12	
	87.5	4.8	1.9420	0.6812	NMNS-cyn 2002-13	
	206	11.5	2.3139	1.0607	NMNS-cyn 2002-14	
	308	16.5	2.4886	1.2175	NMNS-cyn 2003-15	
	228	12	2.3579	1.0792	NMNS-cyn 2003-16	
	434	21	2.6375	1.3222	NMNS-cyn 2003-17	

	241.8	12.8	2.3835	1.1072	NMNS-cyn 2003-18	
	160	8.5	2.2041	0.9294	NMNS-cyn 2003-19	
	264	17	2.4216	1.2304	NMNS-cyn 2003-21	
	197	16	2.2945	1.2041	NMNS-cyn 2003-22	
	182	9	2.2601	0.9542	NMNS-cyn 2003-23	
	249	13.8	2.3962	1.1399	NMNS-cyn 2003-24	
	281	17	2.4487	1.2304	NMNS-cyn 2003-25	
	223	11	2.3483	1.0414	NMNS-cyn 2003-26	
	240	14	2.3802	1.1461	NMNS-cyn 2003-27	
	327	16.5	2.5145	1.2175	NMNS-cyn 2005-01	
	231	12	2.3636	1.0792	NMNS-cyn 2005-02	
	319	18	2.5038	1.2553	NMNS-cyn 2005-03	
	176.5	9.2	2.2467	0.9638	NMNS-cyn 2005-04	
	207.5	11.2	2.3170	1.0492	NMNS-cyn 2005-05	
	284	16	2.4533	1.2041	NMNS-cyn 2005-06	
	211.8	11	2.3259	1.0414	NMNS-cyn 2005-07	
	282	15	2.4502	1.1761	NMNS-cyn 2005-08	
	281	12	2.4487	1.0792	NMNS-cyn 2005-09	
	256	16	2.4082	1.2041	NMNS-cyn 2005-10	
	287	15.8	2.4579	1.1987	NMNS-cyn 2005-11	
	290	16	2.4624	1.2041	NMNS-cyn 2005-12	
	169	8.8	2.2279	0.9445	NMNS-cyn 2005-13	
	231.5	12.2	2.3646	1.0864	NMNS-cyn 2005-14	
	263	14	2.4200	1.1461	NMNS-cyn 2005-15	

	232	13	2.3655	1.1139	NMNS-cyn 2005-16	
Keichousaurus	207	10	2.3160	1.0000	NMNS-cyn 2005-17	
	281	16.5	2.4487	1.2175	NMNS-cyn 2005-18	
	207	11	2.3160	1.0414	NMNS-cyn 2005-19	
	178.5	9	2.2516	0.9542	NMNS-cyn 2005-20	
	268	15.5	2.4281	1.1903	NMNS-cyn 2005-21	
	258	14	2.4116	1.1461	NMNS-cyn 2005-22	
	213	11	2.3284	1.0414	NMNS-cyn 2005-23	
	280	15	2.4472	1.1761	NMNS-cyn 2005-24	
	175	10.5	2.2430	1.0212	NMNS-cyn 2005-25	
	227	11	2.3560	1.0414	NMNS-cyn 2005-26	
	186	10.5	2.2695	1.0212	NMNS-cyn 2005-27	
	281	16.5	2.4487	1.2175	NMNS-cyn 2005-28	
	93	4.8	1.9685	0.6812	NMNS-cyn 2005-29	
	217	11	2.3365	1.0414	NMNS-cyn 2005-30	
	185	10.5	2.2672	1.0212	NMNS-cyn 2005-31	
	224	12	2.3502	1.0792	NMNS-cyn 2005-32	
	65	3.2	1.8129	0.5051	NMNS-kiko 2004-01	
	52.3	3	1.7185	0.4771	NMNS-kiko 2004-03	
	143	8	2.1553	0.9031	NMNS-kiko 2005-X	
	120	6.5	2.0792	0.8129	NMNS-kiko 2005-Y	
	328	17	2.5159	1.2304	NMNS-kiko 2005-XX	
	117.5	6.5	2.0700	0.8129	NMNS-kiko 2005-YY	
	118	7	2.0719	0.8451	NMNS000933-F0034394	

	194	10.5	2.2878	1.0212	NMNS-VL-191	
	335	16.5	2.5250	1.2175	HFUT JYS-15-001	Peraonal observation
	245	13.2	2.3892	1.1206	HFUT JYS-15-002	
	160	8.1	2.2041	0.9085	HFUT JYS-15-011	
Dianopachysaurus	170	9.5	2.2304	0.9777	LPV 31365	Liu et al., 2011[1]
Diandongosaurus	288	16.4	2.4594	1.2148	IVPP V 17761	Shang et al., 2011[138]
	348	18.6	2.5416	1.2695	PKU NMNSS00093-F034398	Sato et al., 2014[139]
	275	16.2	2.4393	1.2095	BGPDB-R0001	Liu et al., 2015[140]
Dianmeisaurus	315	16.1	2.4983	1.2068	IVPP V 18630	Shang et al., 2015[145]
	250	13.5	2.3979	1.1303	IVPP V 17054	Shang et al., 2017[146]
Dawazisaurus	423.8	23	2.6272	1.3617	NMNS000933-F034397	Cheng et al., 2016[147]
Panzhousaurus	344	22.7	2.5366	1.3560	GMPKU-P-1059	Jiang et al., 2019[148]
Yunguisaurus	2000	80	3.3010	1.9031	NMNS 004529/F003862	Sato et al., 2010[70]
	4200	150	3.6232	2.1761	ZMNH M8738	Sato et al, 2013[71]
Wangosauurus	3000	111	3.4771	2.0453	GMPKU-P-1529	Ma et al., 2015[149]
L. sanxiaensis	1496	70	3.1749	1.8451	HFUT YZS-16-01	

Supplementary Table 3. Dataset of geological stage boundaries of 36 taxa with first appearance datum (FAD) and last appearance datum (LAD) in this study (data compiled from references in Supplementary Table 1).

	FAD (Ma)	LAD (Ma)
Araeoscelidia	303.7	272.5
Younginiformes	251.9	247.2
Archosauromorpha	251.9	227
Placodus	247.2	237
Corosaurus	251.2	247.2
Majiashanosaurus	251.2	247.2
Hanosaurus	251.2	247.2
L. buzzii	247.2	237.0
L. hongguoensis	247.2	242.0
L. sanxiaensis	251.2	247.2
L. balsami	242.0	237.0
L. curionii	242.0	237.0
L. valceresii	242.0	237.0
N. marchicus	242.0	237.0
N. yangjuanensis	247.2	242.0
N. mirabilis	242.0	237.0
Paranothosaurus	242.0	237.0
Ceresiosaurus	242.0	237.0

N. jagisteus	242.0	237.0
Simosaurus	242.0	227.0
Germanosaurus	247.2	242.0
N. youngi	242.0	237.0
L. xingyiensis	242.0	237.0
Diandongosaurus	247.2	242.0
Dianopachysaurus	247.2	242.0
Keichousaurus	242.0	232.0
Dactylosaurus	247.2	242.0
Anarosaurus	247.2	242.0
Serpianosaurus	242.0	237.0
Neusticosaurus pusillus	242.0	237.0
Cymatosaurus	247.2	242.0
Augustasaurus	247.2	242.0
Pistosaurus	247.2	242.0
Yunguisaurus	242.0	235
Bobosaurus	237.0	232.0
Plesiosauria	208.5	66.0

Supplementary Table 4. Dataset of geological stage boundaries of 24 eosauropterygian taxa[148] with FAD and LAD (compiled from the references in Supplementary Table 1).

	FAD (Ma)	LAD (Ma)
Hanosaurus	251.2	247.2
Wumengosaurus	247.2	242.0
Qianxisaurus	242.0	237.0
Serpianosaurus-Neusticosaurus	242.0	237.0
Anarosaurus-Dactylosaurus	247.2	242.0
Panzhousaurus	247.2	242.0
Dianopachysaurus	247.2	242.0
Keichousaurus	242.0	232.0
Simosaurus	242.0	227.0
Corosaurus	251.2	247.2
Cymatosaurus	247.2	242
Germanosaurus	247.2	242
Nothosaurus	247.2	237.0
Lariosaurus	247.2	237.0
Wangosaurus	242	235
Yunguisaurus	242	235
Augustasaurus	247.2	242
Pistosaurus	247.2	242

Plesiosaurus	208.5	66
Bobosaurus	237	232
Majiashanosaurus	251.2	247.2
Diandongosaurus	247.2	242
Dianmeisaurus	247.2	242

Supplementary References

1 Liu, J. *et al.* A new pachypleurosaur (Reptilia: Sauropterygia) from the lower Middle Triassic of southwestern China and the phylogenetic relationships of Chinese pachypleurosaurs. *J. Vertebr. Paleontol.* **31**, 292-302 (2011).

2 Rieppel O. 2001 A new species of *Nothosaurus* (Reptilia: Sauropterygia) from the upper Muschelkalk (lower Ladinian) of southwestern Germany. *Palaeontogr. Abt. A.* **263**, 137-161.

3 Benson, R., Evans, M. & Druckenmiller, P. S. High diversity, low disparity and small body size in plesiosaurs (Reptilia, Sauropterygia) from the Triassic–Jurassic boundary. *PLoS ONE* **7**, e31838 (2012).

4 Rieppel, O., Sander, P. M. & Storrs, G. W. The skull of the pistosaur *Augustasaurus* from the Middle Triassic of northwestern Nevada. *J. Vertebr. Paleontol.* **22**, 577-593 (2002).

5 Rieppel, O. & Lin, K. Pachypleurosaurs (Reptilia: Sauropterygia) from the Lower Muschelkalk, and a review of the Pachypleurosauroidea. *Fieldiana (Geology) n.s.* **32**, 1-44 (1995).

6 Rieppel, O. The braincases of *Simosaurus* and *Nothosaurus*: Monophyly of the Nothosauridae (Reptilia: Sauropterygia). *J. Vertebr. Paleontol.* **14**, 9-23 (1994).

7 Cheng, Y. N., Sato, T., Wu, X. C. & Li, C. First Complete Pistosauroid from the Triassic of China *J. Vertebr. Paleontol.* **26**, 501-504 (2006).

8 Rieppel, O. in Encyclopedia of Paleoherpetology Vol. 12A (ed P Wellnhofer) 134 (Verlag Dr. Friedrich Pfeil, Munich, 2000).

9 Zhao, L., Sato, T. & Li, C. The Most Complete Pistosauroid Skeleton from the Triassic of Yunnan, China. *Acta Geol. Sin.- English Edition* **82**, 283-286, (2008).

10 Sato, T., Zhao, L.., Wu, X. C. & Li, C. A new specimen of the Triassic pistosauroid *Yunguisaurus*, with implications for the origin of Plesiosauria (Reptilia, Sauropterygia). *Palaeontology* **57**, 55–76 (2013)

11 Klein, N. Skull morphology of *Anarosaurus heterodontus* (Reptilia: Sauropterygia: Pachypleurosauria) from the Lower Muschelkalk of the Germanic Basin (Winterswijk, The Netherlands). *J. Vertebr. Paleontol.* **29**, 665-676 (2009)

12 Klein, N. Postcranial morphology and growth of the pachypleurosaur *Anarosaurus heterodontus* (Sauropterygia) from the Lower Muschelkalk of Winterswijk, The Netherlands. *Paläontologische Zeitschrift* **86**, 389-408 (2012)

13 Shang, Q., Sato, T., Li, C. & Wu, X. C. New osteological information from a 'juvenile' specimen of *Yunguisaurus* (Sauropterygia; Pistosauroidea). *Palaeoworld* **26**, 500-509 (2017).

14 Gregory, J. T. Osteology and relationships of *Trilophosaurus*. *University of Texas Publication* **4401**, 273-359 (1945).

15 Demar, R. & Bolt, J. R. Dentitional organization and function in a Triassic reptile. *J. Paleontol.* **55**, 967-984 (1981).

16 Heckert, A. B., Lucas, S. G., Kahle, R. & Zeigler, K. New occurrence of *Trilophosaurus* (Repitilia: Archosauromorpha) from the Upper Triassic of West Texas and its biochronological significance. In *Geology of the Llano Estacado: New Mexico Geological Society 52th Cofference Guidebook* (eds. Lucas, S. G., Ulmer-Scholle, D.) 115-122 (2001)

17 Spielmann, J. A., Heckert, A. B. & Lucas, S. G. The Late Triassic archosauromorph *Trilophosaurus* as an arboreal climber. *Riv. Ital. Paleontol. S* **111**, 395-412 (2005).

18 Heckert, A. B. *et al.* Revision of the archosauromorph reptile *Trilophosaurus*, with a description of the first skull of *Trilophosaurus jacobsi*, from the Upper Triassic Chinle Group, West Texas, USA. *Palaeontology* **49**, 621-640 (2006).

19 Mueller, B. D. & Parker, W. G. A new species of *Trilophosaurus* (Diapsida: Archosauromorpha) from the Sonsela Member (Chinle Formation) of Petrified Forest National Park, Arizona. *Museum of Northern Arizona, Bulletin* **62**, 119-125 (2006).

20 Spielmann, J. A., Lucas, S. G., Heckert, A. B., Rinehart, L. F. & Hunt, A. Taxonomy and biostratigraphy of the Late Triassic archosauromorph *Trilophosaurus*. *New Mexico Museum of Natural History and Science Bulletin* **40**, 231-240 (2007).

21 Nesbitt, S. J. *et al.* Postcranial osteology of *Azendohsaurus madagaskarensis* (?Middle to Upper Triassic, Isalo Group, Madagascar) and its systematic position among stem archosaur reptiles. *B. Am. Mus. Nat. Hist.* **398**, 1-126 (2015).

22 Peyer, B. Die Triasfauna der Tessiner Kalkalpen. XII. *Macrocnemus bassanii* Nopcsa. *Abhandlungen der schweizerischen paläontologischen Gesellschaft* **59**, 1-140 (1937).

23 Kuhn-Schnyder, E. Ein weiterer Schädel von *Macrocnemus bassanii* Nopcsa aus der anisischen Stufe der Trias des Monte San Giorgio (Kt. Tessin, Schweiz). *Palaeontol. Z.* **36**, 110-133 (1962).

24 Rieppel, O. & Gronowske, R. W. The loss of the lower temporal arcade in diapsid reptiles. *Zool. J. Linn. Soc-Lond.* **72**, 203-217 (1981).

25 Rieppel, O. The hind limd of *Macronemus bassanii* (Nopcsa) (Reptilia, Diapsida): deverlopment and functional anatomy. *J. Vertebr. Paleontol.* **9**, 373-387 (1989).

26 Li, C., Zhao, L. & Wang, L. A new species of *Macrocnemus* (Reptilia: Protorosauria) from the Middle Triassic of southwestern China and its palaeogeographical implication. *Science in Sci. China Ser. D.* **50**, 1601-1605 (2007).

27 Zhang, B., Chen, X. & Cheng, L. Discovery of *Macrocnemus* cf. *fuyuanensis* of the Middle Triassic in Xinyi, Guizhou Province, SW China. *Geology and Mineral Resources of South China* **2**, 43-47 (2010).

28 Jiang, D. *et al.* New information on the protorosaurian reptile *Macrocnemus fuyuanensis* Li et al., 2007, from the Middle/Upper Triassic of Yunnan, China. *J. Vertebr. Paleontol.* **31**, 1230-1237 (2011).

29 Fraser, N. C. & Furrer, H. A new species of *Macrocnemus* from the Middle Triassic of the eastern Swiss Alps. *Swiss J. Geosci.* **106**, 199-206 (2013).

30 Pritchard, A. C., Turner, A. H., Nesbitt, S. J., Irmis, R. B. & Smith, N. D. Late Triassic tanystropheids (Reptilia, Archosauromorpha) from northern New Mexico (Petrified Forest Member, Chinle Formation) and the biogeography, functional morphology, and evolution of Tanystropheidae. *J. Vertebr. Paleontol.* **35**, e911186; 10.1080/02724634.2014.911186 (2015).

31 Renesto, S. *Megalancosaurus*, a possibly arboreal archosauromorph (Reptilia) from the Upper Triassic of Northern Italy. *J. Vertebr. Paleontol.* **14**, 38-52 (1994).

32 Renesto, S. & Dalla Vecchia, F. M. The unusual dentition and feeding habits of the prolacertiform reptile *Langobardisaurus* (Late Triassic, Northern Italy). *J. Vertebr. Paleontol.* **20**, 622-627 (2000).

33 Renesto, S., Dalla Vecchia, F. M. & Peters, D. Morphological evidence for bipedalism in the Late Triassic prolacertiform reptile *Langobardisaurus*. *Senckembergiana Lethaea* **82**, 95-106 (2002).

34 Saller, F., Renesto, S. & Dalla Vecchia, F. M. First record of *Langobardisaurus* (Diapsida, Protorosauria) from the Norian (Late Triassic) of Austria, and a revision of the genus. *Neues Jahrb. Geol. Palaontol. Abh.* **268**, 83-95 (2013).

35 Peyer, B. Die Triasfauna der Tessiner Kalkalpen. II. *Tanystropheus longobardicus* Bass. sp. *Abhandlungen der schweizerischen paläontologischen Gesellschaft* **50**, 6-110 (1931).

36 Wild, R. Die Triasfauna der Tessiner Kalkalpen. XXIII. *Tanystropheus longobardicus* (Bassani) (Neue Ergebnisse). *Schweizerische Paläontologische Abhandlungen* **95**, 1-162 (1973).

37 Wild, R. Die Triasfauna der Tessiner Kalkalpen XXIV. Neue Funde von *Tanystropheus* (Reptilia, Squamata). *Schweizerische Paläontologische Abhandlungen* **102**, 1-43 (1980).

38 Wild, R. *Tanystropheus* (Reptilia: Squamata) and its importance for stratigraphy. *Mémoires de la Société Géologique de France NS* **139**, 201-206 (1980).

39 Wild, R. & Oosterink, H. *Tanystropheus* (Reptilia, Squamata) aus dem Unteren Muschelkalk von Winterswijk, Holland. *Grondboor en Hamer* **5**, 142-148 (1984).

40 Dalla Vecchia, F. M. *Tanystropheus* (Archosauromorpha, Prolacertiformes) remains from the Triassic of the Northern Friuli (NE Italy). *Riv. Ital. Paleontol. S.* **106**, 135-140 (2000).

41 Renesto, S. A new specimen of *Tanystropheus* (Reptilia Protorosauria) from the Middle Triassic of Switzerland and the ecology of the genus. *Rivi. Ital. Paleontol. S.* **111**, 377-394 (2005)

42 Dalla Vecchia, F. M. Resti di *Tanystropheus*, saurotterigi e "rauisuchi" (Reptilia) nel Triassico medio della Val Aupa (Moggio Udinese, Udine). *Gortania. Atti Mus. Friul. St. Nat.* **27**, 25-48 (2006).

43 Li, C. A juvenile *Tanystropheus* sp. (Protorosauria, Tanystropheidae) from the Middle Triassic of Guizhou, China. *Vertebr. PalAsiat.* **45**, 37-42 (2007).

44 Nosotti, S. *Tanystropheus longobardicus* (Reptilia, Protorosauria): re-interpretations of the anatomy based on new specimens from the Middle Triassic of Besano (Lombardy, Northern Italy). *Memorie della Società Italiana di Scienze Naturali e del Museo Civico di Storia Naturale di Milano Memorie* **35**, 1-88 (2007).

45 Rieppel, O. *et al. Tanystropheus* cf. *T. longobardicus* from the early Late Triassic of Guizhou Province, southwestern China. *J. Vertebr. Paleontol.* **30**, 1082-1089 (2010).

46 Li, C. First record of protorosaurid reptile (Order Protorosauria) from the Middle Triassic of China. *Acta Geol. Sin.* **77**, 419-423 (2003).

47 Li, C., Rieppel, O. & LaBarbera, M. C. A Triassic aquatic protorosaur with an extremely long neck. *Science* **305**, 1931-1931 (2004).

48 Rieppel, O., Li, C. & Fraser, N. C. The skeletal anatomy of the triassic protorosaur *Dinocephalosaurus orientalis* Li, from the Middle Triassic of Guizhou Province, southern China. *J. Vertebr. Paleontol.* **28**, 95-110 (2008).

49 Liu, J., Organ, C. L., Benton, M. J., Brandley, M. C. & Aitchison, J. C. Live birth in an archosauromorph reptile. *Nat. Commun.* **8**, 10.1038/ncomms14445 (2017).

50 Seeley, H. G. Researches on the structure, organization, and classification of the fossil Reptilia. I. On *Protorosaurus speneri* (von Meyer). *Philos. T. Roy. Soc. B.* **178**, 187-213 (1887).
51 Romer, A. S. The relationships of the Permian reptile *Protorosaurus*. *Am. J. Sci.* **245**, 19-30 (1947).
52 Evans, S. E. & King, M. S. A new specimen of *Protorosaurus* (Reptilia: Diapsida) from the Marl Slate (late Permian) of Britain. *Proceedings of the Yorkshire Geological and Polytechnic Society* **49**, 229-234 (1993).
53 Gottmann-Quesada, A. & Sander, P. M. A redescription of the early archosauromorph *Protorosaurus speneri* Meyer,1832,and its phylogenetic relationships. *Palaeontogr. Abt. A.* **287**, 123-220 (2009).
54 Jalil, N. E. A new prolacertiform diapsid from the Triassic of North Africa and the interrelationships of the Prolacertiformes. *J. Vertebr. Paleontol.* **17**, 506-525 (1997).
55 Rieppel, O. In *The genus Placodus : systematics, morphology, paleobiogeography, and paleobiology*. Vol. n.s. no.31 (ed.)1-44 (Fieldiana, 1995).
56 Meyer, H. v. Die Placodonten, eine Familie von Sauriern der Trias. *Palaeontographica* **11**, 175-221 (1863).
57 Broili, F. Zur Osteologie des Schadels von *Placodus. palaeontographica* **59**, 147-155 (1912).
58 Rieppel, O. Paraplacodus and the phylogeny of the Placodontia (Reptilia: Sauropterygia). *Zool. J. Linn. Soc-Lond.* **130**, 635-659 (2000).
59 Jiang, D. *et al.* First record of Placodontoidea (Reptilia, Sauropterygia, Placodontia) from the Eastern Tethys. *J. Vertebr. Paleontol.* **28**, 904-908 (2008).
60 Diedrich, C. G. Palaeoecology of *Placodus gigas* (Reptilia) and other placodontids--Middle Triassic macroalgae feeders in the Germanic Basin of central Europe--and evidence for convergent evolution with Sirenia. *Paleogeogr. Paleoclimatol. Paleoecol.* **285**, 287-306 (2010).
61 Storrs, G. W. *Anatomy and relationships of Corosaurus alcovensis (Reptilia: Nothosauria) and the Triassic Alcova Limestone of Wyoming*, Yale University, (1986).
62 Kuhn-Schnyder, E. Die Wirbeltierfauna der Trias der Tessiner Kalkalpen. *Geol. Rundsch.* **53**, 393-412 (1964).
63 Rieppel, O. *Corosaurus alcovensis* case and the phylogenetic interrelationships of Triassic stem-group Sauropterygia. *Zool. J. Linn. Soc-Lond.* **124**, 1-41 (1998).
64 Schrammen, A. Beitrag zur Kenntniss der Nothosauriden des unteren Muschelkalkes in Oberschlesien. *Zeitschrift der Deutschen Geologischen Gesellschaft* **51**, 388-408 (1899).
65 Huene, E. v. *Cymatosaurus* und seine Beziehungen zu anderen Sauropterygiern. *Neues. Jahrb. Geol. P-M.* **1944**, 192-222 (1944).
66 Rieppel, O. Revision of the sauropterygian reptile genus *Cymatosaurus* v. Fritsch, 1894, and the relationships of *Germanosaurus* Nopcsa, 1928, from the Middle Triassic of Europe. 1-38 (Chicago, Field Museum of Natural History, 1997).
67 Maisch, M. W. W. A well preserved skull of *Cymatosaurus* (Reptilia: Sauropterygia) from the uppermost Buntsandstein (Middle Triassic) of Germany. *Neues Jahrb. Geol. Palaontol. Abh.* **272**, 213-224 (2014).
68 Dalla Vecchia, F. M. A new sauropterygian reptile with plesiosaurian affinity from the Late Triassic of Italy. *Riv. Ital. Paleontol. S.* **112**, 207-225 (2006).

69 Fabbri, M., Dalla Vecchia, F. M. & Cau, A. New information on *Bobosaurus forojuliensis* (Reptilia: Sauropterygia): implications for plesiosaurian evolution. *Hist. Biol.* **26**, 661-669 (2014).
70 Sato, T., Cheng, Y. N., Wu, X. C. & Li, C. Osteology of *Yunguisaurus* Cheng et al., 2006 (Reptilia; Sauropterygia), a Triassic pistosauroid from China. *Paleontol. Res.* **14**, 179-195 (2010).
71 Sato, T., Zhao, L., Wu, X. C. & Li, C. A new specimen of the Triassic pistosauroid *Yunguisaurus*, with implications for the origin of Plesiosauria (Reptilia, Sauropterygia). *Palaeontology* **57**, 55-76 (2013).
72 Sander, P. M., Rieppel, O. & Bucher, H. A new pistosaurid (Reptilia: Sauropterygia) from the Middle Triassic of Nevada and its implications for the origin of the plesiosaurs. *J. Vertebr. Paleontol.* **17**, 526-533 (1997).
73 Rieppel, O., Sander, P. M. & Storrs, G. W. The skull of the pistosaur *Augustasaurus* from the Middle Triassic of northwestern Nevada. *J. Vertebr. Paleontol.* **22**, 577-592 (2002).
74 Meyer, H. v. Mittheilung, an Professor Bronn gerichtet. *Neues Jahrbuch fur Mineralogie, Geognosie, Geologie und Petrefaktenkunde*, 559-560 (1839).
75 Meyer, H. v. Die Saurier des Muschelkalkes mit Rücksicht auf die Saurier aus buntem Sandstein und Keuper. *Zur Fauna der Vorwelt* **2**, 1-167 (1847-55).
76 Sues, H. D. Postcranial skeleton of Pistosaurus and interrelationships of the Sauropterygia (Diapsida). *Zoological Journal of the Linnean Society* **90**, 109-131 (1987).
77 Huene, F. v. Neue Beobachtungen an *Simosaurus*. *Acta Zoologica, Stockholm* **1921**, 201-239 (1921).
78 Rieppel, O. Osteology of *Simosaurus gaillardoti* and the relationships of stem-group Sauropterygia. *Fieldiana (Geology) n.s.* **28**, 1-85 (1994).
79 Rieppel, O. Sauropterygia I. in *Encyclopedia of Paleoherpetology*, *Volume 12A*, (ed. Wellnhofer, P) 1-134 (Verlag Dr. Friedrich Pfeil, Munich, 2000).
80 Dalla Vecchia, F. M. First record of *Simosaurus* (Sauropterygia, Nothosauroidea) from the Carnian (Late Triassic) of Italy. *Riv. Ital. Paleontol. S.* **114**, 273-285 (2008).
81 Gürich, G. J. E. Über einen neuen Nothosaurus von Gogolin, Oberschlesien. *Zeitschrift der deutschen Geologischen Gesellschaft* **43**, 967-970 (1891).
82 Koken, E. Beiträge zur Kenntniss der Gattung Nothosaurns. *Zeitschrift der deutschen geologischen Gesellschaft* **1**, 337-377 (1893).
83 Nopcsa, F. Palaeontological notes on reptiles. *Geologica Hungarica, Series Palaeontologica* **1**, 3-84 (1928).
84 Peyer, B. Die Triasfauna der Tessiner Kalkalpen. IV. *Ceresiosaurus calcagnii* nov. gen. nov. spec. *Abhandlungen der Schweizerischen Paldontologischen Gesellschaft* **51**, 1-68 (1931).
85 Hänni, K. *Die Gattung Ceresiosaurus: Ceresiosaurus calcagnii Peyer und Ceresiosaurus lanzi* n. sp. (Lariosauridae, Sauropterygia) 1-147 (vdf Hochschulverlag AG, 2004).
86 Quesada, J. M. & Aguera González, S. Descripción del primer ejemplar de *Ceresiosaurus* (Reptilia: Sauropterygia), hallado en la Península Ibérica en el Ladiniense (Triásico medio) de Mont-ral-Alcover (Tarragona). *Estud. Geol.* **61**, 247-269 (2005).
87 Curioni, G. Cenni sopra un nuovo saurio fossile dei monti di Perledo suI Lario e suI terreno che lo racchiude. **16**, 159-170 *Gior. Ist. Lombardo Sci. Lett.* (1847).

88 Boulenger, G. A. On a nothosaurian reptile from the Trias of Lombardy, apparently referable to *Lariosaurus*. *Transactions of the Zoological Society of London* **14**, 1-10 (1896).
89 Bassani, F. Sui fossili e sull'età degli schisti bituminosi triasici di Besano in Lombardia. *Atti della Società Italiana di Scienze Naturali e del Museo Civico di Storia Naturale* **29**, 15-72 (1886).
90 Mariani, E. Su un nuovo esemplare di *Lariosaurus balsami* Cur. trovato negli scisti di Perledo sopra Varenna (Lago di Como). *Atti della Societa Italiana di Scienze Naturali e del Museo Civico di Storia Naturale di Milano* **62**, 218-225 (1923).
91 Zapfe, H. & Konig, H. Neue Reptilienfunde aus der Mitteltrias der Gailtaler Alpen (Kärnten, Österreich). *Sitzungsberichte der Osterreichischen Akademie der Wissenschaften, mathematisch-naturwissenschaftliche Klasse, Abt. I,* **189**, 65-82 (1980).
92 Ticli, B. Due esemplari di *Lariosaurus balsami* Curioni presenti nei Musei Civici di Lecc. *Natura (Milano)* **75**, 69-74 (1984).
93 Mazin, J. M. A specimen of *Lariosaurus balsami* Curioni 1847, from the eastern Pyrenees (France). *Palaeontogr. Abt. A.* **189**, 159-169 (1985).
94 Kuhn-Schnyder, E. Die Triasfauna der Tessiner Kalkalpen. XXVI. *Lariosaurus lavizzarii* n. sp. (Reptilia, Sauropterygia). *Schweizerische Paläontologische Abhandlungen* **110**, 1-24 (1987).
95 Renesto, S. A juvenile *Lariosaurus* (Reptilia, Sauropterygia) from the Kalkschieferzone (uppermost Ladinian) near Viggiu' (Varese, Northern Italy). *Riv. Ital. Paleontol. S.* **99**, 199-210 (1993).
96 Rieppel, O. *Lariosaurus balsami* Curioni (Reptilia, Sauropterygia) aus den Gailtaler Alpen. *Carinthia II* **104**, 345-356 (1994).
97 Tschanz, K. *Lariosaurus Buzzii* N. Sp. from the Middle Triassic of Monte San Giorgio (Switzerland) with Comments on the Classification of *Nothosaurs*. *Palaeontogr. Abt. A.* **208,** 153-179 (1989).
98 Rieppel, O. The status of the sauropterygian reptile genera *Ceresiosaurus*, *Lariosaurus* and *Silvestrosaurus* from the Middle Triassic of Europe. *Fieldiana (Geology) n.s.* **38**, 1-46 (1998).
99 Jiang, D., Maisch, M. W., Sun, Z., Sun, Y. & Hao, W. A new species of *Lariosaurus* (Reptilia, Sauropterygia) from the Middle Anisian (Middle Triassic) of southwestern China. *Neues Jahrb. Geol. und Palaontol. Abh.* **242**, 19-42 (2006).
100 Tintori, A. & Renesto, S. A new *Lariosaurus* from the Kalkschieferzone (Uppermost Ladinian) of Valceresio (Varese, N. Italy). *B. Soc. Paleontol. Ital.* **29**, 197-210 (1990).
101 Renesto, S., Pareo, M. & Lombardo, C. A new specimen of the sauropterygian reptile *Lariosaurus* from the Kalkschieferzone (Uppermost Ladinian) of Valceresio (Varese, N Italy). *Neues. Jahrb. Geol. P-M.* **2004**, 351-369 (2004).
102 Li, J., Liu, J. & Rieppel, O. A new species of *Lariosaurus* (Sauropterygia: Nothosauridae) from Triassic of Guizhou, southwest China. *Vertebr. PalAsiat.* **40**, 114-126 (2002).
103 Lin, W. *et al.* A new specimen of *Lariosaurus xingyiensis* (Reptilia, Sauropterygia) from the Ladinian (Middle Triassic) Zhuganpo Member, Falang Formation, Guizhou, China. *J. Vertebr. Paleontol.* e1278703; 10.1080/02724634.2017.1278703 (2017).
104 Edinger, T. Über *Nothosaurus* I. *Senckenbergiana* **3**, 121-129 (1921).

105 Rieppel, O. & Wild, R. A Revision of the Genus *Nothosaurus* (Reptilia: Sauropterygia) from the Germanic Triassic, with comments on the status of *Conchiosaurus clavatus*. *Fieldiana (Geology) n.s.* **34**, 1-82 (1996).
106 Münster, G. Vorlaunge Nachricht über einige neue Reptilien im Muschelkalke von Baiern. *Neues Jahrbuch für Mineralogie, Geognosie, Geologie und Petrefaktenkunde* **1834**, 521-527 (1834).
107 Quenstedt, F. A. *Handbuch der Petrefaktenkunde*. 1-792 (H. Laupp, 1852).
108 Berckhemer, F. Bericht der Wurtt. Naturaliensammlung in Stuttgart: Geologische Abteilung. *Jahreshefte des Vereins fur vaterlandische Naturkunde in Wurttemberg* **86**, xxi-xxviii (1930).
109 Rieppel, O. The status of the nothosaurian reptile *Elmosaurus lelmensis*, with comments on *Nothosaurus mirabilis*. *Palaeontology* **36**, 967-974 (1993).
110 Rieppel, O. A new species of *Nothosaurus* (Reptilia: Sauropterygia) from the upper Muschelkalk (lower Ladinian) of southwestern Germany. *Palaeontogr. Abt. A.* **263**, 137-161 (2001).
111 Peyer, B. Die Triasfauna der Tessiner Kalkalpen. XIV. *Paranothosaurus amsleri* nov. gen. nov. spec. *Abhandlungen der schweizerischen paläontologischen Gesellschaft* **62**, 1-87 (1939).
112 Kuhn-Schnyder, E. Der Schädel von *Paranothosaurus amsleri* Peyer aus dem Grenzbitumenhorizont der anisisch-ladinischen Stufe der Trias des Monte San Giorgio (Kt. Tessin, Schweiz). *Eclogae. Geol. Helv.* **59**, 517-540 (1966).
113 Jiang, D., Maisch, M. W., Hao, W., Sun, Y. & Sun, Z. *Nothosaurus yangjuanensis* n. sp (Reptilia, Sauropterygia, Nothosauridae) from the middle Anisian (Middle Triassic) of Guizhou, southwestern China. *Neues. Jahrb. Geol. P-M.* **2006**, 257-276 (2006).
114 Shang, Q. A new species of *Nothosaurus* from the early Middle Triassic of Guizhou, China. *Vertebr. PalAsiat.* **44**, 237-249 (2006).
115 Yin, C., Hao, W., Sun, Z., Sun, Y. & Jiang, D. New material of *Nothosaurus yangjuanensis* from the Middle Anisian (Middle Triassic) of Guizhou Province, souwestern China. *Acta Scientiarum Naturalium Universitatis Pekinensis* **50**, 467-475 (2014).
116 Li, J. & Rieppel, O. A new nothosaur from Middle Triassic of Guizhou, China. *Vertebr. PalAsiat.* **42**, 1-12 (2004).
117 Ji, C. *et al.* A new specimen of *Nothosaurus youngi* from the Middle Triassic of Guizhou, China. *J. Vertebr. Paleontol.* **34**, 465-470 (2014).
118 Dames, W. *Anarosaurus pumilio* nov. gen. nov. sp. *Zeitschrift der deutschen geologischen Gesellschaft*, 74-85 (1890).
119 Rieppel, O. The status of *Anarosaurus multidentatus* von Huene (Reptilia, Sauropterygia), from the lower Anisian of the Lechtaler Alps (Arlberg, Austria). *Paläntologische Zeitschrift* **69**, 289-299 (1995).
120 Gürich, G. Ueber einige Saurier des oberschlesischen Muschelkalkes. *Zeitschrift der deutschen geologischen Gesellschaft* 125-144 (1884).
121 Sues, H. D. & Carroll, R. L. The pachypleurosaurid *Dactylosaurus schroederi* (Diapsida: Sauropterygia). *Can J Earth Sci* **22**, 1602-1608 (1985).
122 Rieppel, O. Status of the pachypleurosauroid *Psilotrachelosaurus toeplitschi* Nopcsa (Reptilia, Sauropterygia), from the Middle Triassic of Austria. *Fieldiana (Geology) n.s.* **27**, 1-17 (1993).

123 Seeley, H. G. On *Neusticosaurus pusillus* (Fraas), an Amphibious Reptile having Affinities with the Terrestrial Nothosauria and with the Marine Plesiosauria. *Quarterly Journal of the Geological Society* **38**, 350-366 (1882).

124 Pinna, G. La collezione di rettili triassici di Besano (Varese) del Museo Civico di Storia Naturale di Milano. *Natura* **58**, 177-192 (1967).

125 Carroll, R. L. & Gaskill, P. The nothosaur *Pachypleurosaurus* and the origin of plesiosaurs. *Philos. T. Roy. Soc. B* **309**, 343-393 (1985).

126 Sander, P. M. The pachypleurosaurids (Reptilia: Nothosauria) from the Middle Triassic of Monte San Giorgio (Switzerland) with the description of a new species. *Philos. T. Roy. Soc. B* **325**, 561-666 (1989).

127 Renesto, S. Peculiar preservation of a juvenile pachypleurosaurid from Besano (Italy). *Riv. Ital. Paleontol. S.* **112**, 373-382 (2006).

128 Rieppel, O. A new pachypleurosaur (Reptilia: Sauropterygia) from the Middle Triassic of Monte San Giorgio, Switzerland. *Philos. T. Roy. Soc. B* **323**, 1-73 (1989).

129 Rieppel, O. & Hagdorn, H. in *Ancient Marine Reptiles* (eds Callaway, J. & Nicholls, E.) 121-144 (Academic Press, 1997).

130 Hugi, J. & Scheyer, T. M. Ossification sequences and associated ontogenetic changes in the bone histology of pachypleurosaurids from Monte San Giorgio (Switzerland/Italy). *J. Vertebr. Paleontol.* **32**, 315-327 (2012).

131 Beardmore, S. R., Orr, P. J., Manzocchi, T., Furrer, H. & Johnson, C. Death, decay and disarticulation: Modelling the skeletal taphonomy of marine reptiles demonstrated using Serpianosaurus (Reptilia; Sauropterygia). *Palaeogeogr. Palaeoclimatol. Palaeoecol.* **337**, 1-13 (2012).

132 Young, C. C. On the new pachypleurosauroidea from Keichow, South-West China. *Vertebr. PalAsiat.* **2**, 69-81 (1958).

133 Lin, K. & Rieppel, O. Functional morphology and ontogeny of *Keichousaurus hui* (Reptilia, Sauropterygia). *Fieldiana (Geology) n.s.* **39**, 1-39 (1998).

134 Holmes, R., Cheng, Y. N. & Wu, X. C. New information on the skull of *Keichousaurus hui* (Reptilia: Sauropterygia) with comments on sauropterygian interrelationships. *J. Vertebr. Paleontol.* **28**, 76-84 (2008).

135 Cheng, Y. N., Holmes, R., Wu, X. C. & Alfonso, N. Sexual dimorphism and life history of *Keichousaurus hui* (Reptilia: sauropterygia). *J. Vertebr. Paleontol.* **29**, 401-408 (2009).

136 Motani, R., Jiang, D., Rieppel, O., Xue, Y. & Tintori, A. Adult sex ratio, sexual dimorphism and sexual selection in a Mesozoic reptile. *Proc. R. Soc. B* **282**, 20151658; 10.1098/rspb.2015.1658 (2015).

137 Xue, Y. *et al.* New information on sexual dimorphism and allometric growth in *Keichousaurus hui*, a pachypleurosaur from the Middle Triassic of Guizhou, South China. *Acta. Palaeontol. Pol.* **60**, 681-687 (2015).

138 Shang, Q., Wu, X. C. & Li, C. A new eosauropterygian from Middle Triassic of eastern Yunnan Province, southwestern China. *Vertebr. Palasiat.* **49**, 155-171 (2011).

139 Sato, T., Cheng, Y. N., Wu, X. C. & Shan, H. *Diandongosaurus acutidentatus* Shang, Wu & Li, 2011 (Diapsida: Sauropterygia) and the relationships of Chinese eosauropterygians. *Geol. Mag.* **151**, 121-133 (2014).

140 Liu, X. *et al.* A new specimen of *Diandongosaurus acutidentatus* (Sauropterygia) from the Middle Triassic of Yunnan,China. *Vertebr. Palasiat.* (2015).

141 Young, C. C. & Dong, Z. M. In *Aquatic Reptiles from the Triassic of China*. Vol. 9 (ed. Young, C. C. & Dong, Z. M.) 1-53 (Academia Sinica, 1972).

142 Rieppel, O. The systematic status of *Hanosaurus hupehensis* (Reptilia, Sauropterygia) from the Triassic of China. *J. Vertebr. Paleontol.* **18**, 545-557 (1998).

143 Jiang, D. *et al.* The Early Triassic eosauropterygian Majiashanosaurus discocoracoidis, gen. et sp. nov. (Reptilia, Sauropterygia), from Chaohu, Anhui Province, People's Republic of China. *J. Vertebr. Paleontol.* **34**, 1044-1052,

144 Wintrich, T., Hayashi, S., Houssaye, A., Nakajima, Y. & Sander, P. M. A Triassic plesiosaurian skeleton and bone histology inform on evolution of a unique body plan. *Sci. Adv.* **3**, (2017).

145 Shang, Q. & Li, C. A new small-sized eosauropterygian (Diapsida: Sauropterygia) from the Middle Triassic of Luoping, Yunnan, southwestern China. *Vertebr. PalAsiat.* **53**, 265-280 (2015).

146 Shang, Q., Li, C. & Wu, X. C. New information on *Dianmeisaurus gracilis* Shang & Li, 2015. *Vertebr. Palasiat.* **55,** 145-160 (2017).

147 Cheng, Y. N., Wu, X. C., Sato, T. & Shan, H. Y. *Dawazisaurus brevis*, a new eosauropterygian from the Middle Triassic of Yunnan, China. *Acta Geol. Sin. - English Edition* **90**, 401-424, (2016).

148 Jiang, D., Lin, W., Rieppel, O., Motani, R. & Sun, Z. A new Anisian (Middle Triassic) eosauropterygian (Reptilia, Sauropterygia) from Panzhou, Guizhou Province, China. *J. Vertebr. Paleontol.* **38**, 1480113; 10.1080/02724634.2018.1480113 (2019).

149 Ma, L., Jiang, D., Rieppel, O., Motani, R. & Tintori, A. A new pistosauroid (Reptilia, Sauropterygia) from the late Ladinian Xingyi marine reptile level, southwestern China. *J. Vertebr. Paleontol.* **35**, e881832; 10.1080/02724634.2014.881832 (2015).
