# Supplementary material for: An Early Triassic sauropterygian and associated fauna from South China provide insights into Triassic ecosystem health
Source: Commun Biol. 2020 Feb 11;3:63. doi: 10.1038/s42003-020-0778-7 (PMC7012838; doi:10.1038/s42003-020-0778-7)
Supplement: Supplementary file 2 — Description of Additional Supplementary Files [file 42003_2020_778_MOESM2_ESM.docx]

**Supplementary Data 1.**

The novel data matrix compiled in this study. The file was constructed using NDE Version 0.5.0 and contains 36 relevant taxa and 181 characters in total.
