# Supplementary material for: An Early Triassic sauropterygian and associated fauna from South China provide insights into Triassic ecosystem health
Source: Commun Biol. 2020 Feb 11;3:63. doi: 10.1038/s42003-020-0778-7 (PMC7012838; doi:10.1038/s42003-020-0778-7)
Supplement: Supplementary file 4 — Reporting Summary [file 42003_2020_778_MOESM4_ESM.pdf]

nature research

Corresponding author(s): Jun Liu

Last updated by author(s): Dec 20, 2019

Reporting Summary

Nature Research wishes to improve the reproducibility of the work that we publish. This form provides structure for consistency and transparency in reporting. For further information on Nature Research policies, see Authors & Referees and the Editorial Policy Checklist.

Statistics

For all statistical analyses, confirm that the following items are present in the figure legend, table legend, main text, or Methods section.

n/a	Confirmed	
☐	☒	The exact sample size (*n*) for each experimental group/condition, given as a discrete number and unit of measurement
☐	☒	A statement on whether measurements were taken from distinct samples or whether the same sample was measured repeatedly
☐	☒	The statistical test(s) used AND whether they are one- or two-sided<br>*Only common tests should be described solely by name; describe more complex techniques in the Methods section.*
☒	☐	A description of all covariates tested
☐	☒	A description of any assumptions or corrections, such as tests of normality and adjustment for multiple comparisons
☒	☐	A full description of the statistical parameters including central tendency (e.g. means) or other basic estimates (e.g. regression coefficient) AND variation (e.g. standard deviation) or associated estimates of uncertainty (e.g. confidence intervals)
☒	☐	For null hypothesis testing, the test statistic (e.g. *F*, *t*, *r*) with confidence intervals, effect sizes, degrees of freedom and *P* value noted<br>*Give P values as exact values whenever suitable.*
☒	☐	For Bayesian analysis, information on the choice of priors and Markov chain Monte Carlo settings
☒	☐	For hierarchical and complex designs, identification of the appropriate level for tests and full reporting of outcomes
☐	☒	Estimates of effect sizes (e.g. Cohen's *d*, Pearson's *r*), indicating how they were calculated

Our web collection on statistics for biologists contains articles on many of the points above.

Software and code

Policy information about availability of computer code

Data collection	No software was used.
Data analysis	The character list and data matrix were constructed using NDE Version 0.5.0. Phylogenetic analysis was performed using the software PAUP Version 4.0a 164 for Windows. The StratPhyloCongruence function of Strap package in R was applied to calculate MSM* and GER.

For manuscripts utilizing custom algorithms or software that are central to the research but not yet described in published literature, software must be made available to editors/reviewers. We strongly encourage code deposition in a community repository (e.g. GitHub). See the Nature Research guidelines for submitting code & software for further information.

Data

Policy information about availability of data

All manuscripts must include a data availability statement. This statement should provide the following information, where applicable:

- Accession codes, unique identifiers, or web links for publicly available datasets
- A list of figures that have associated raw data
- A description of any restrictions on data availability

We declare that the datasets supporting this article have been uploaded as part of the supplementary material.

Field-specific reporting

Please select the one below that is the best fit for your research. If you are not sure, read the appropriate sections before making your selection.

☒ Life sciences ☐ Behavioural & social sciences ☐ Ecological, evolutionary & environmental sciences

October 2018

Life sciences study design

All studies must disclose on these points even when the disclosure is negative.

Sample size	We described one fossil specimen in detail. To analyze the phylogenetic relationships of the newly reported taxon, we included all of the eosauropterygian taxa for which at least one of the referred specimens has been examined by us in person and several relevant outgroups into our data matrix. This leads to 36 taxa in the data matrix. Via the comparative study, 44 more or less new characters were added into an existing morphological data matrix. This results in a new data matrix containing 181 characters in total. In total, 268 specimens were referred for constructing the new data matrix. In order to estimate the body length of the new specimen, all of the 103 published complete specimens of Triassic eosauropterygians have been used for extrapolation.
Data exclusions	No data were excluded.
Replication	The bootstrap analysis has been replicated for 1000 times. The number of permutations for resampled trees and the randomly generated trees in the significance tests in stratigraphic congruence are 1000 times.
Randomization	Not relevant.
Blinding	Not relevant.

Reporting for specific materials, systems and methods

We require information from authors about some types of materials, experimental systems and methods used in many studies. Here, indicate whether each material, system or method listed is relevant to your study. If you are not sure if a list item applies to your research, read the appropriate section before selecting a response.

Materials & experimental systems

n/a	Involved in the study	
☒	☐	Antibodies
☒	☐	Eukaryotic cell lines
☐	☒	Palaeontology
☒	☐	Animals and other organisms
☒	☐	Human research participants
☒	☐	Clinical data

Methods

n/a	Involved in the study	
☒	☐	ChIP-seq
☒	☐	Flow cytometry
☒	☐	MRI-based neuroimaging

Palaeontology

Specimen provenance	The specimen was collected from a then active limestone quarry in the southwestern side of Yingzishan hill, Yuan'an County, Hubei Province in 2008. No permit was needed when the specimen was collected according to the relevant regulations available then.
Specimen deposition	The specimen is deposited in the Paleontological Lab of Hefei University of Technology, Hefei, China.
Dating methods	No new dates are provided.

☒ Tick this box to confirm that the raw and calibrated dates are available in the paper or in Supplementary Information.
